# Electroosmotic flow: From microfluidics to nanofluidics

**DOI:** 10.1002/elps.202000313

**Published:** 2021-01-22

**Authors:** Amer Alizadeh, Wei‐Lun Hsu, Moran Wang, Hirofumi Daiguji

**Affiliations:** ^1^ Department of Mechanical Engineering The University of Tokyo Tokyo Japan; ^2^ Department of Engineering Mechanics Tsinghua University Beijing P. R. China

**Keywords:** Electrical double layer / Electro osmosis / Microchannels / Nanochannels / Porous media

## Abstract

Electroosmotic flow (EOF), a consequence of an imposed electric field onto an electrolyte solution in the tangential direction of a charged surface, has emerged as an important phenomenon in electrokinetic transport at the micro/nanoscale. Because of their ability to efficiently pump liquids in miniaturized systems without incorporating any mechanical parts, electroosmotic methods for fluid pumping have been adopted in versatile applications—from biotechnology to environmental science. To understand the electrokinetic pumping mechanism, it is crucial to identify the role of an ionically polarized layer, the so‐called electrical double layer (EDL), which forms in the vicinity of a charged solid–liquid interface, as well as the characteristic length scale of the conducting media. Therefore, in this tutorial review, we summarize the development of electrical double layer models from a historical point of view to elucidate the interplay and configuration of water molecules and ions in the vicinity of a solid–liquid interface. Moreover, we discuss the physicochemical phenomena owing to the interaction of electrical double layer when the characteristic length of the conducting media is decreased from the microscale to the nanoscale. Finally, we highlight the pioneering studies and the most recent works on electro osmotic flow devoted to both theoretical and experimental aspects.

**Abbreviations**
AC‐EO: AC electroosmosis
BS: basic Stern
BL: buffer layer
EDL: electrical double layer
ETL: electrical triple layer
EOI: electroosmosis instability
EKR: electrokinetic remediation
GC‐EDL: Gouy‐Chapman electrical double layer
HS: Helmholtz–Smoluchowski
ICEO: induced charge electroosmosis
IREOF: induced reverse EOF
LBM: lattice Boltzmann method
MD: molecular dynamics
NS: Navier–Stokes
NP: Nernst–Planck
NPS: nonporous silica
ODS: octadecyl silyl
OHP: outer‐Helmholtz plane
PTT: Phan‐Thien‐Tanner
SC: surface conductance
TIC: transport‐induced‐charge
VEDL: viscoelectric double layer
ZP: zeta potential plane

## 1 Introduction

Ever since Ferdinand Friedrich Reuss reported for the first time, more than two centuries ago, his interesting observation of moving water through a plug of clay upon application of an external electric field, a huge body of theoretical and experimental studies have been carried out based upon the discovery. The most important outcome of this discovery was the ability to make water flow without any mechanical parts and solely through application of an external electric field. The working principle of this concept, which was later called electrokinetic transport, involves the interplay of the external electric field and a charged interface that is neutralized by a counter‐charge layer of the liquid. One of the well‐known categories of electrokinetic transport is EOF. EOF, which can be employed as a pump to drive water from one side to the other side of an electrically charged medium, has been extensively used and studied [1, 2, 3, 4, 5, 6, 7, 8, 9, 10, 11]. The key role in this pumping phenomenon is played by the electrically charged solid–liquid interface, the so‐called electrical double layer (EDL). The EDL, which is a composition of charged solid surface and a very tiny layer of counter‐charges in an aqueous solution (around a few nanometers), was first investigated in the 19th century [12], and has been further studied in the 20th century [13, 14, 15] and in contemporary times [16, 17, 18, 19, 20]. Consequently, it is of utmost importance to enhance our understanding of the configuration of water and ionic species in the vicinity of an electrically charged solid surface.

One important characteristic feature of EOF is that its volume flow rate can be comparable to that of pressure‐driven flow in micro‐ and nanoscale media [21]. Therefore, it is important to study EOF in micro‐ and nanochannels/porous media and understand the impact on the flow of water or charged species. It is worth pointing out that the characteristic size of the medium has a considerable impact on EOF due to the overlapping of EDLs. Let us imagine that the height of a channel is comparable with the EDL thickness. Thus, it is expected that the EDLs interact. This interaction of the EDLs makes a major part of the channel electrically non‐neutral [22, 23]. As a result, due to the electrostatic forces, the channel will become selective to the counter‐ions, repelling most of the coions. This interesting phenomenon has been the basis of many practical applications—from biophysics [24] to electrokinetic remediation (EKR) of contaminants from underground water resources [25, 26].

This tutorial aims at reviewing EOF at the microfluidic and nanofluidic scale. We start from a short historical review of EOF and the story of its discovery (Section 2). After discussing the background, we present and discuss the evolution of the various EDL models (Section 2.1). After understanding how a solution in the vicinity of a charged solid surface will be configured and a chemically active solid surface will be electrically charged, we will then discuss EOF through microchannels (Section 3.1) and microporous media (Section 3.2). Next, we will turn our interest down to nanoscale channels (Section 4.1) and nanoporous media (Section 4.2), which we will address both theoretically and experimentally. We present out conclusions in .

## 2 History and background

**Figure 1:**
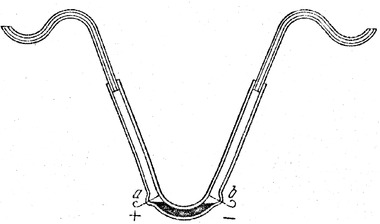
Reproduced schematic diagram [28] of the experiments done by Reuss from his article [28]. The experiments conducted in a U‐type glass tube filled with water and the lower part was filled with insoluble particles, such as sandstone, which created a porous barrier. Reuss observed that when an external voltage was applied to the water, it began to pass through the porous barrier from the anode (+) to the cathode (−) side.

When German scientist Ferdinand Friedrich Reuss reported his observation of water flowing through a plug of clay under application of an external electrical voltage through the two ends of a U‐type tube, he presented his discovery as a lecture entitled “Notice on a new, hitherto unknown effect of galvanic electricity” to the Physico‐Medical Society of Moscow in 1807 [27, 28]. Subsequently, he published two papers in Latin and French [29, 30] to describe this unknown phenomenon in detail. In his first experiment (Fig. 1), he observed the flow of water (electroosmosis) through a wetted porous barrier made of powdered quartz. Upon application of an external electric field, the water on the cathode (−) side was raised while that on the anode (+) side descended. After 2 h, he observed that the entire cathode side of the U‐tube, together with the S‐type glass tube, was filled with water while the anode side of the U‐tube was discharged.

**Figure 2:**
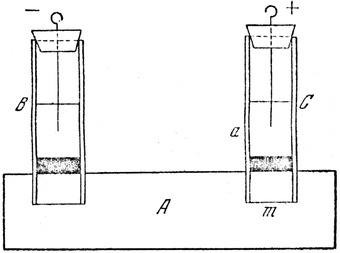
Reproduced schematic diagram of Reuss’ second experiment [28, 30]. Reuss inserted two glass tubes into a block of moist clay (part A) and applied an external electric field to the water inside the glass tubes.

Reuss conducted his second experiment [30] in which two glass tubes were filled with water and a thin layer of well‐washed sand was kept at the bottom (Fig. 2). In this setup, Reuss observed that by applying an external electric field, the sand at the bottom of the positive tube swelled upward and tended to penetrate the sand layer (demonstrated as label A in Fig. 2). With the benefit of hindsight, we today will interpret this phenomenon as electrophoretic movement of the charged sand particles. However, Reuss interpreted this observation as EOF into the sand.

Reuss made several false deductions while trying to interpret this phenomenon. He stated that the liquid between the poles of a battery is continuously driven from the positive pole toward the negative pole. He also believed that the presence of porous barriers makes this movement visible by counteracting the impact of gravity [28]. While some previous works supported his conclusions [31, 32], later in this article we will show that according to our modern understanding, these conclusions are false. Biscombe [28] has reviewed the history of EOF discovery, which can be referred for further details.

### 2.1 Models to explain the charged solid–liquid interface

As we mentioned in the last section, Reuss concluded that the fluid flow must be due to the application of an external electric field in an aqueous solution. However, the main physics underlying this phenomenon was a mystery to him. He was not aware of the impact of the solid–liquid interface on it. In 1859, Georg Quincke [33] conducted several important experiments that proved to be a great step forward in shedding light on Reuss’ discovery [32]. He conducted the reverse electroosmosis experiment by applying a pressure gradient. In his setup, the pumped water went through a tube where he measured a potential difference. He found that adding sodium chloride lowered the measured electric potential. The main conclusion from his experiments was that the sign of the electric potential is independent of the water flow rate, tube diameter, the concentration of the dissolved ions, and even the porous barrier material in which he employed the glass, sand, graphite, silk, and ivory. Here, we should note that, in his experiments, the measured electric potential was essentially changed by utilizing different materials. Quincke's experiments led him to the idea that there must be an excess space charge rather than the surface charge. This hypothesis was a remarkable milestone for the following works that attempted to explain the underlying physics of the earlier experiments.

**Figure 3:**
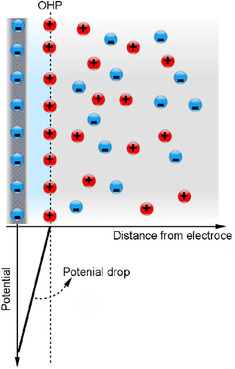
Schematic illustration of the Helmholtz EDL model. The surface is negatively charged and positive ions (counter‐ions) are attracted to a location called the Helmholtz plane. In this model, the net electric charge density of the ions is zero for the solution beyond the Helmholtz plane.

The pioneering theoretical attempt was done by the German physicist and physician Herman von Helmholtz in 1850 [12]. Helmholtz was the first who paid attention to the nature of the electrode–electrolyte or charged solid–electrolyte interface. Essentially, a charged interface will attract free counter‐ions in the solution and repel coions because of the Columbic forces. The charged interface, along the layer of counter‐ions that are attracted to the interface, was called the EDL. Helmholtz assumed that there would be no electron transfer at the charged interface and that the ions were solid spherical particles with a specific diameter. According to the Helmholtz model, as illustrated in Fig. 3, the charge that holds (electrode) or is acquired on the solid–liquid interface must be balanced by the redistribution of ions arranged in a parallel hypothetical plane with the solid–liquid interface.

The development of the kinetic theory of molecular behavior proved that the Helmholtz EDL model was not realistic [34]. The main shortcoming of the Helmholtz model was in ignoring the thermal motion of the counter‐ions and the possibility of adsorption onto the solid surface. In the Helmholtz model, the thickness of the Helmholtz double layer is independent of the thermochemical properties of the solution.

**Figure 4:**
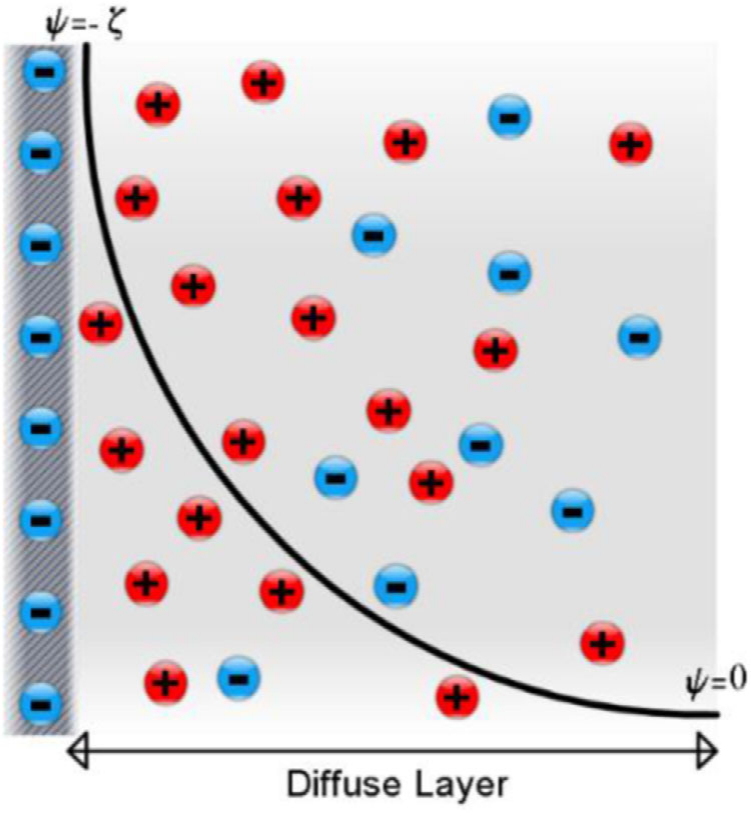
Gouy–Chapman illustration of the ionic distribution and electric potential distribution in the vicinity of a charged solid surface.

Taking into account the thermal motion of the ionic species, it is reasonable to say that the attracted counter‐ions in the vicinity of the solid–liquid interface will spread out in space [21]. The spreading of the counter‐ions into the bulk solution forms a *diffuse layer* that, contrary to the Helmholtz double layer, varies with the thermochemical properties of the solution. The idea of such a diffuse layer was independently proposed by French physicist Louis Georges Gouy (1910) and English physical chemist David Leonard Chapman (1913). The resulting model is now named after them as the Gouy–Chapman electrical double layer (GC‐EDL) model. According to GC‐EDL (Fig. 4) model, the electric potential decreases exponentially with the distance from the charged solid–liquid interface. The thickness of the region with nonzero net electric charge density ( $\rho_{e}=\;e\left(c^{+}-c^{-}\right)$ where e denotes the electron charge and $c^{+}$ and $c^{-}$ are the counter‐ion and co‐ion concentration, respectively), depends on the bulk ionic concentration that, for instance, in the solution would be stretched over 100 nm in a very dilute electrolyte solution.

The standard solution of the GC‐EDL model, which is based on statistical mechanics, can be found in textbooks as early as Adamson [35] and Overbeek [36]. The GC‐EDL model is based on the Poisson equation, which is solved from the solid surface (at x=0, ψ=−ζ) to the bulk solution (where x→∞ and ψ=0) and is written as

(1) $$\left(\nabla^{2}\psi \right)=-\frac{\rho_{e}}{\varepsilon_{0}\varepsilon_{r}}=-\frac{e\left(c^{+}-c^{-}\right)}{\varepsilon_{0}\varepsilon_{r}},$$

where ψ, $\varepsilon_{0}$, $\varepsilon_{r}$ denote the electric potential, vacuum electrical permittivity, and the solution's relative permittivity with respect to vacuum, respectively. For the ionic species in the solution, it is reasonable to say that they feel the local electrostatic potential from the charged solid–liquid interface. Consequently, we can introduce the Boltzmann equation as

(2) $$c_{i}=c_{i}^{b}\exp\left(-\frac{z_{i}\psi }{k_{B}T}\right),$$

where $c_{i}$, $c_{i}^{b}$, $z_{i}$, $k_{B}$, and T represent the *i*th ionic concentration, the bulk ion concentration, the ionic valence (e.g., for a monovalent ionic solution $z_{1}=\;+1$ and $z_{2}=\;-1$), Boltzmann constant ( $k_{B}=\;1.380649\;\times 10^{-23}$ J/K), and the absolute temperature of the solution, respectively. Introducing Eq. (2) into Eq. (1) gives rise to the Poisson–Boltzmann equation:

(3) $$\left(\varepsilon_{0}\varepsilon_{r}\right)\;\left(\nabla^{2}\psi \right)=2ec_{i}^{b}\sinh\left(\frac{e\psi }{k_{B}T}\right).$$

The right‐hand side of Eq. (3) can be simplified if we assume that $\psi \ll \frac{k_{B}T}{e}$. The term $V_{t}=k_{B}\;T/e$ is well‐known as the thermal potential and provides a measure of the induced potential energy on an elementary charge (i.e., electron charge) [37]. At room temperature, $V_{t}$ is about 25 mV. Recalling the assumption for the local electric potential in solution, if ψ≪25mV, then Eq. (3) can be simplified as

(4) $$\left(\varepsilon_{0}\varepsilon_{r}\right)\left(\nabla^{2}\psi \right)\approx 2ec_{i}^{b}\left(\frac{e\psi }{k_{B}T}\right),$$

which linearizes the Poisson–Boltzmann equation. Historically, Debye and Hückel simply extended the exponential term related to the Boltzmann equation as truncated Taylor series to the first order [38]. In the Taylor series, the zeroth‐order vanishes because the whole system is electroneutral ($\sum_{i}^{n}z_{i}ec_{i}=\;0$). However, the first order leaves the Helmholtz‐type equation. Rewriting Eq. (4) by only keeping the Laplace term at the left‐hand side, we finally obtain

(5) $$\left(\nabla^{2}\psi \right)\approx \left(\frac{2e^{2}c_{i}^{b}}{\varepsilon_{0}\varepsilon_{r}k_{B}T}\right)\psi .$$

Considering the right‐hand side of Eq. (5), the term $\kappa \;=\sqrt{\left(\frac{2e^{2}c_{i}^{b}}{\varepsilon_{0}\varepsilon_{r}k_{B}T}\right)}\;$ has got the inverse of a length, which is attributed to the characteristic length of the EDL and called the Debye length. Consequently, Eq. (5) can be rewritten as

(6) $$\left(\nabla^{2}\psi \right)\approx \kappa^{2}\psi .$$

It is worth pointing out that κ is also referred as the Debye–Hückel parameter. Solving Eq. (6) together with the Boltzmann equation (Eq. (2)) determines the distribution of the ionic species and the electric potential in the vicinity of a charged solid–liquid interface. Regarding the solution for Eq. (6), we can subject the equation to the following boundary conditions:

(7) $$\begin{array}{c} \begin{array}{c} a)\;\mathrm{At}\;y=H,\frac{d\psi }{dy}=0\left(\mathrm{due}\;\mathrm{to}\;\mathrm{symmetry}\;\mathrm{at}\;\mathrm{center}\;\mathrm{of}\;\mathrm{channel}\right),\\ b)\;\mathrm{At}\;y=0,\;\psi =\zeta \left(\mathrm{potential}\;\mathrm{at}\;\mathrm{shear}\;\mathrm{plane}\right), \end{array}\;\;\; \end{array}$$

where the *y* direction represents the normal direction to the solid–liquid interface. Thus, the solution for Eq. (6) is [21]

(8) $$\psi \;=\;\zeta \frac{\cosh\left(\kappa y\right)}{\cosh\left(\kappa H\right)}.$$

In Eq. (8), *H* represents the distance far from a single solid–liquid interface or in the middle of a 2D micro‐ or nanochannel. Here, it is worth pointing that we can also propose an analytical solution for the Poisson–Boltzmann equation (Gouy–Chapman theory, Eq. (3)) without any approximation (Debye–Hückel). It should be noted that the GC‐EDL model is a nonlinear theory for ionic distribution in the vicinity of the solid–liquid interface. In this regard, the analytical solution for Eq. (3) can be

(9) $$\Psi \;=\ln\left(\frac{1+\exp\left(-\kappa y\right)\tanh\left(\frac{\Psi_{s}}{4}\right)}{1-\exp\left(-\kappa y\right)\tanh\left(\frac{\Psi_{s}}{4}\right)}\right),$$

where Ψ denotes the nondimensional form of ψ defined as $\Psi \;=\frac{\psi }{V_{t}}\;$; at x=0 and x→∞ we have $\Psi \;=\Psi_{s}\;$ and Ψ=0, respectively. There are several excellent textbooks that can be referred for further study about the electrostatic interaction between planar and curved surfaces [21, 39].

**Figure 5:**
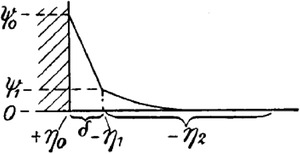
Reproduced sketch of the Stern EDL model [41] where the solid surface is assumed to be positively charged. In his model, the first layer, called Stern layer, has the thickness represented by δ, and the diffuse layer assumed to be beyond the Stern layer.

Following the Helmholtz and GC‐EDL models, researchers attempted to propose more complicated EDL models that could tackle the shortcomings of these previous models. For instance, the Gouy–Chapman model suffers from a lack of generality for highly charged solid–liquid interfaces. According to the GC‐EDL model, if we increase zeta potential to a very high value, the distributed ionic concentration is predicted to be infinitely large. This prediction originates from assuming the ionic species as point charges (no ionic volume effect). However, in reality, the ionic species have nonzero volume that approaches the solid surface to a distance not less than their Stokes radii or the effective hydrated radius in solution [40]. Thus, the German‐American physicist Otto Stern (1924) proposed a combination of the Helmholtz and GC‐EDL models to overcome the shortcomings of both models [41]. He suggested that the EDL in the solution consists of two layers. The first layer is a stagnant layer of hydrated ions (Helmholtz model) and the second layer is a diffuse layer of the ionic species (GC model). Figure 5 shows the reproduced sketch of the Stern EDL model from his paper [41]. According to his model, the electric potential changes linearly from the solid surface ($\psi_{0}$ or $\psi_{s}$) to a Stern plane potential ($\psi_{1}$ or $\psi_{d}$). The ions inside the Stern layer are assumed to be immobile, while the ions beyond the Stern layer are assumed to be point charges (according to the GC‐EDL model). It is worth noting that the distribution in the diffuse layer of the Stern model is determined via the Boltzmann distribution equation.

The boundary between the immobile (Stern layer) and mobile (diffuse layer) layer in the Stern model is assumed to be the shear plane in which the *zeta potential* (ζ) represents the electric potential on this plane. The real position of the shear plane has been the subject of long debates, which is out of scope of the present work [17, 42, 43, 44, 45, 46].

Although the Stern EDL model is considered a significant step forward in our understanding of the surface charge at the solid–liquid interface, it still deficient in several aspects, such as ignoring the chemical reactions that take place on the solid–liquid interface, the solution pH effect, and, importantly, it does not provide a method to calculate the surface charge and zeta potential based on the solution pH and ion concentration.

Approximately 50 years after Stern, Davis et al. [47] attempted to explain and determine the surface charge and electric potential on the Stern EDL model by proposing a detailed surface complexation model for oxide surfaces. They showed that the surface charge is dominated by surface complex formation of ionizable sites and electrolyte ions. However, for a very dilute solution, the surface ionization is significantly related to the concentration of the hydronium ($H^{+}$) and hydroxyl ($OH^{-}$). The remarkable conclusion of their model is that the electrolyte ions and the hydronium and hydroxyl concentrations are working jointly to determine the surface charge and electric potential via chemical reaction with the surface sites. Their novel results proposed that adsorption density, surface charge, and zeta potential could be estimated simultaneously. The surface of the chemically active materials, such as quartz, clay, etc. not only could be simply ionized but also the surface complexes formed because of the association and dissociation of the hydronium and hydroxyl ions from the solid surface [48].

**Figure 6:**
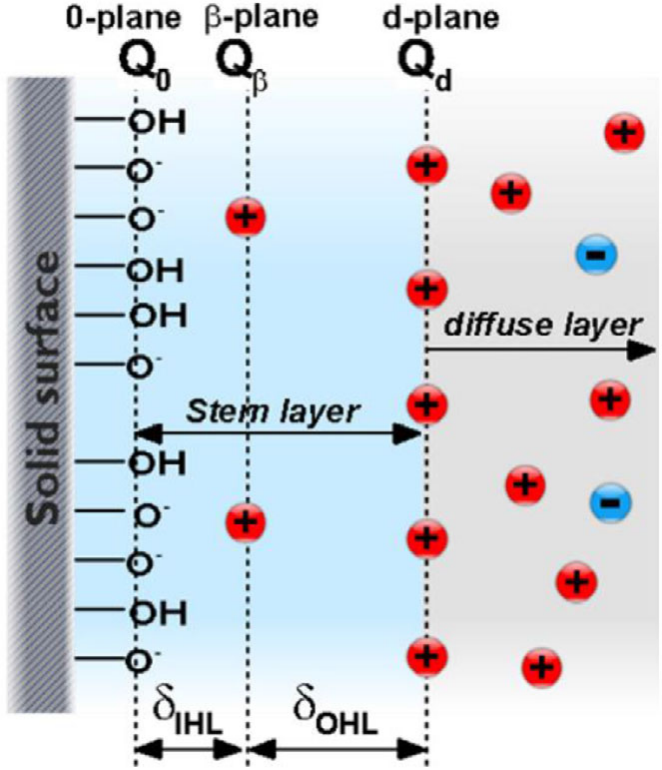
Configuration of the ETL model. In this model, it is assumed that the ions could be distributed in three layers that lie on two planes near the charged surface. The 0‐plane, β‐plane, and d‐plane are the inner‐Helmholtz plane, outer‐Helmholtz plane, and the starting edge of the diffuse layer, respectively [47].

In this model, it is assumed that the Stern layer consists of two layers, both belonging to the immobile part of the EDL. The complex of the Stern layer with the diffuse layer is called the electrical triple layer (ETL) model. Figure 6 depicts the configuration of the ETL model, in which the Stern layer is divided into two layers via the inner‐ and outer‐Helmholtz planes (OHPs) following the Stern–Grahame electrostatic capacitor model of the inner region [45, 48]. In this model, it is assumed that the OHP coincides with the starting edge of the diffuse layer or, in other words, the shear plane.

According to the model that Davis [47] proposed, which was later developed by Kitamura et al. [15], the contribution of the salt‐ion adsorption to the surface charge on mineral surfaces is based on the following four chemical reactions [49]:

(10) $${\mathrm{SiOH}}_{2}^{+}⇌\mathrm{SiOH}+H^{+},{\;K}_{a1}^{\mathrm{int}},$$

(11) $$\mathrm{SiOH}⇌\mathrm{Si}O^{-}+H^{+},{\;K}_{a2}^{\mathrm{int}},$$

(12) $$\mathrm{Si}O^{-}+M^{+}⇌\mathrm{SiOM},{\;K}_{M}^{\mathrm{int}},$$

(13) $${\mathrm{SiOH}}_{2}^{+}+A^{-}⇌\mathrm{SiO}H_{2}A,{\;K}_{A}^{\mathrm{int}},$$

where $K^{\mathrm{int}}$ in each equation represents the associated equilibrium constants for the reactions. Equations (10) and (11) explain the impact of the hydronium concentration. If the concentration of $H^{+}$ increases, the chemical reactions (10) and (11) will move to the left side. Similarly, the impact of the metal ionic species (cation M^+^ and anion A^−^) is explained by Eqs. (12) and (13). It is noteworthy that in the ETL model, the protonation rate of the siloxane group is very low for the pH range of 3–9. As a result, we can ignore the fourth reaction (Eq. (13)) in the set of ETL equations described above. In the ETL model, the surface charge and electric potential on the three planes are calculated by considering the continuity equation for the surface charge density on the solid surface, the Grahame equation [45], and the differential capacitance of the inner‐ and outer‐Helmholtz layers.

**Figure 7:**
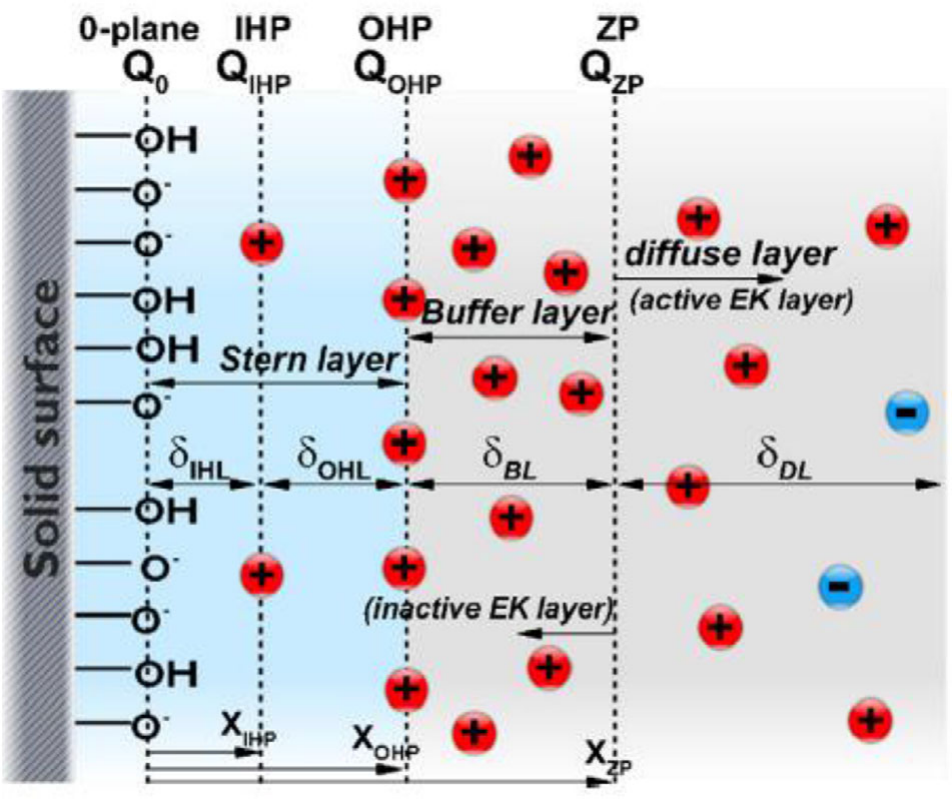
Electrical quad‐layer model, in which a new layer was added between the zeta potential plane and the OHP, namely the BL. The figure has been reprinted from [17]. The positions of different planes are shown by X and the thickness of each layer is represented by δ.

Although the ETL model proved to be a great step forward in figuring out the surface charge and electric potential at the chemically active solid surface and the aqueous solution interface based on the properties of the solid surface, it cannot explain some experimental observations such as the impact of solution temperature on the zeta potential [50] or the concentration‐dependent electrical conductivity of ultra‐narrow channels (approximately 2 nm) [17]. With this aim, Alizadeh et al. [17] further developed the ETL model in which an extra layer was added between the zeta potential (ZP) plane and the OHP, called the buffer layer (BL) (Fig. 7). The significant property of this layer is that its position from the solid surface has a flexible nature, which is a function of the bulk ion concentration and solution temperature. In their work, they showed that the zeta potential plane (ZP) recedes to the bulk solution or approaches the solid surface when the bulk ion concentration decreases or increases, respectively.

**Figure 8:**
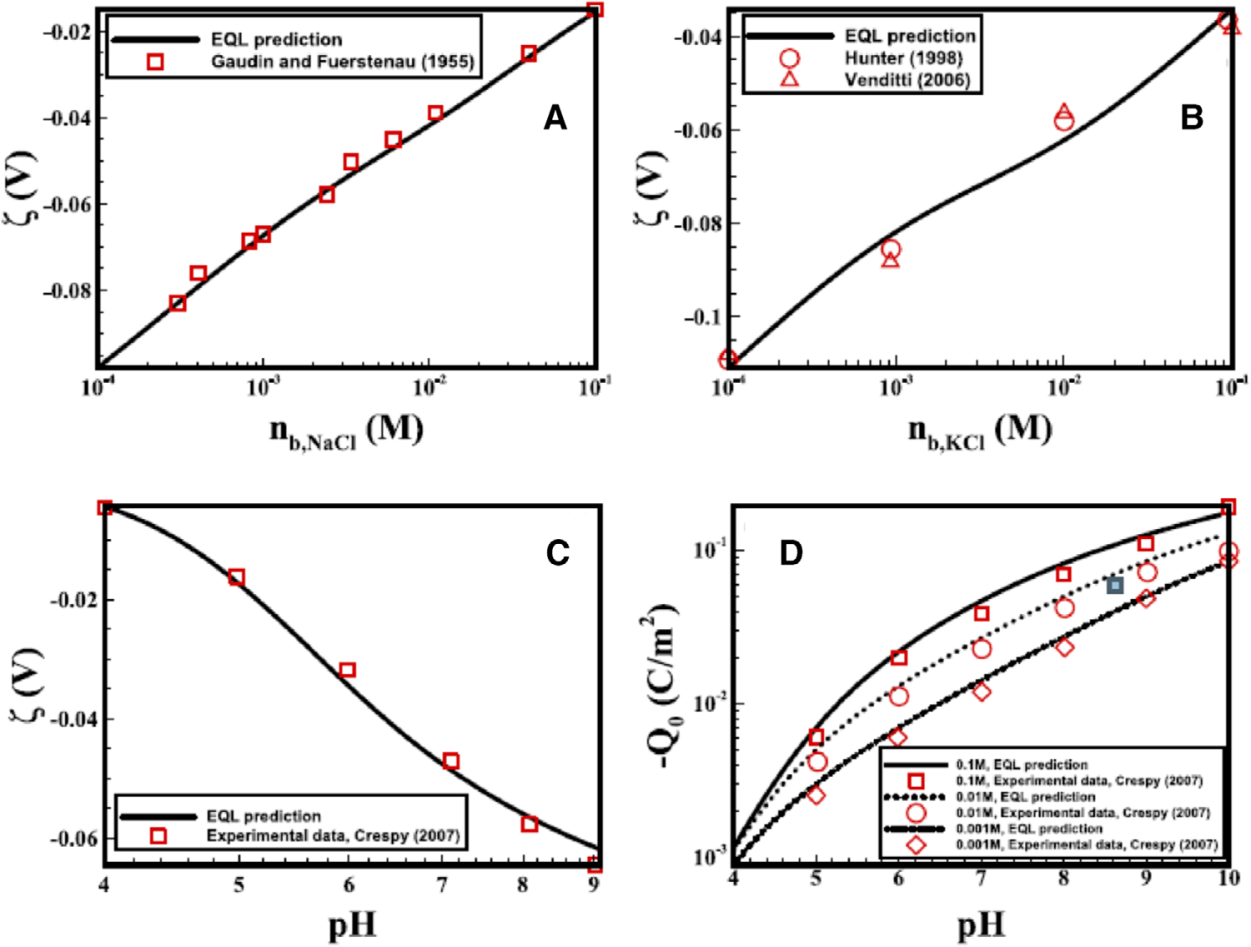
Zeta potential versus bulk ion concentration for (A) NaCl and (B) KCl. (C) zeta potential and (D) surface charge versus the solution pH. The figures have been reprinted from [17].

The same chemical reactions were considered as mentioned above for ETL (Eqs. (10)‐(13)). The BL properties were integrated into the model by assuming a new capacitance, which was defined as

(14) $$\psi_{OHP}-\;\psi_{ZP}=-\frac{Q_{ZP}}{C_{BL}},$$

where $C_{BL}$, $Q_{ZP}$, and ψ represent BL capacitance, the surface charge on the starting edge of the diffuse layer, and the electric potential on the OHP and ZP, respectively. It is worth noting that the BL capacitance is

(15) $$C_{BL}=\frac{\varepsilon_{0}\varepsilon_{r}}{\delta_{BL}}\;,$$

where $\varepsilon_{0}\varepsilon_{r}$ is the aqueous solution electrical permittivity; and $\delta_{BL}$ is the BL thickness, which is a fitting parameter. To determine the thickness of BL, one needs to employ the ab‐initio methods (i.e., molecular dynamics [MD] simulations) to gain a deeper understanding of this layer. However, as the aim of Alizadeh et al. [17] work was to present a macroscopic model, the thickness of BL was defined as a fitting parameter. They demonstrated that the electrical quad‐layer model could predict the measured zeta potentials and surface charge (Fig. 8) as a function of bulk solution properties (i.e., bulk ionic concentration and solution pH).

The intense electric field within the electric double layer could significantly alter the physical properties of the water solvent from its bulk values. Specifically, the orientation of water molecules in the vicinity of the charged surface favors hydrogen bonding interactions, thereby increasing the shear force in the flow direction normal to the surface. From an activation energy perspective, the imposed electric field decreases the vibration frequency of molecules giving rise to an increase in the activity energy $E_{a}$ and, as such, an increase in the apparent viscosity (according to Reynolds’ equation for the viscosity of liquids μ, where the coefficient A is constant):

(16) $$\mu =A\;\exp\left(\frac{E_{a}}{k_{B}T}\right),$$

In 1939, Andrade and Dodd were the first to experimentally demonstrate this phenomenon for polar solvents by measuring the flow rate of a pressure‐driven flow through a slit channel with an applied electric field normal to the flow direction [51, 52]. They observed that in the case of polar solvents, the apparent viscosity increased considerably with the magnitude of the applied electric field whereas it remained constant for nonpolar solvents. In a subsequent study published in 1950, they quantified this phenomenon and revealed that the increase in viscosity is a function of the applied magnitude of electric field, as seen in Eq. (17) [53]:

(17) $$\mu =\mu_{0}\;\exp\left(f\left|E^{2}\right|\right),$$

where $\mu_{0}$ is the viscosity in the absence of an electric field, E is the local electric field, and $f\;=\;\Delta E_{a}/\left(k_{B}T\right)$ is referred to as the viscoelectric coefficient, in which $\Delta E_{a}$ is the activation energy variation due to the presence of E.

At low applied electric fields, that is, $f|E{|}^{2}\ll 1$, the higher order terms in the Maclaurin expansion of the exponential function in Eq. (17) become negligible, resulting in a simplified viscoelectric equation, which shows that the increase of viscosity is proportional to the square of the local electric field:

(18) $$\mu =\mu_{0}\;\left(1+f\left|E^{2}\right|\right).$$

According to Debye and Onsager's theories, f is subject to the degree of polarization of the solvent molecules, which can be expressed as

(19) $$f\propto \frac{\tau^{2}}{3k_{B}T}\left(\frac{3\varepsilon_{r}}{2\varepsilon_{r}+1}\right),$$

where τ is the dipole moment. In 1961, Lyklema and Overbeek analyzed f for water based on a polarization theory of spherical molecules [42]. Using an average value of structural coefficients from previous data of organic polar solvents, f for water was estimated to be 10.2 × 10^−16^ m^2^V^−2^. This theoretical estimate was later verified by Hunter and Leyendekkers in 1978, who experimentally obtained a slightly lower f = (5∼10) × 10^−16^ m^2^V^−2^ for water between two parallel sheets [54].

**Figure 9:**
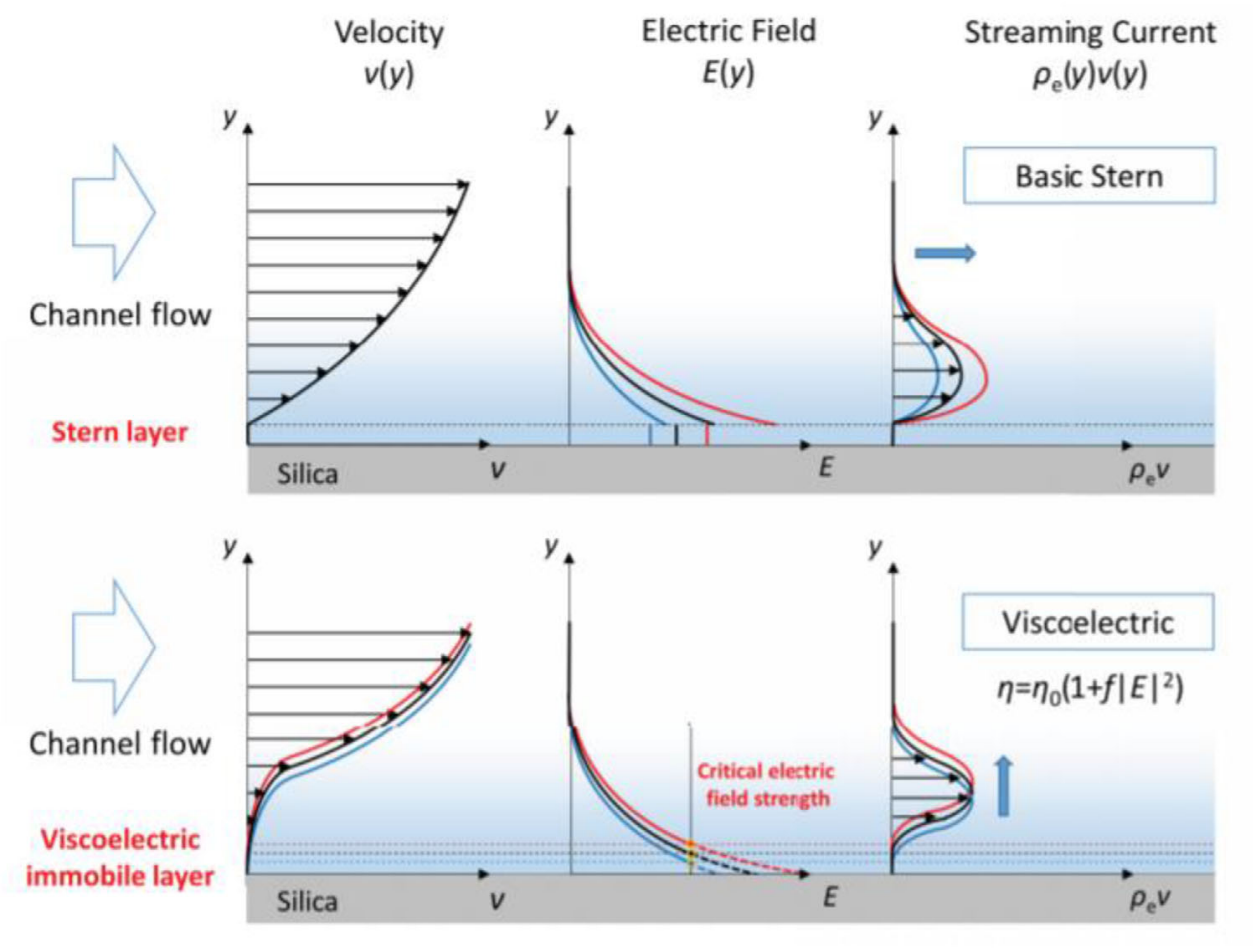
Schematic of the velocity, electric field, and streaming current profiles in the vicinity of the surface at high concentrations under the basic Stern (BS) and viscoelectric double layer (VEDL) models, respectively. *v*, *E*, and *ρ*_e_ denote the velocity, electric field in EDL, and space charge density, respectively. The figure has been reprinted from [18].

Hsu et al. [18] considered viscoelectric effects in EDL models via computational simulations, following the viscoelectic coefficient estimated by Lyklema and Overbeek, and quantitatively obtained experimental measurements of four classical electrokinetic phenomena: electrophoresis, electroosmosis, streaming potential, and streaming current [18]. They reached a consistent conclusion for streaming potential and streaming current: the measured electrokinetic quantities are independent of the surface charge (potential) as the electric field at the interface is higher than a critical value. As seen in Fig. 9, as opposed to models that assume a constant viscosity (e.g., the basic Stern (BS) model, which predicts that the streaming current will increase with the increase in surface charge), the viscoelectric double layer model (VEDL) shows that the streaming current is insensitive to the surface conditions at high surface charge densities due to the presence of a viscoelectric immobile layer. This implies that when the surface charge is high, the surface charge conditions may not be accurately measured by electrokinetic methods.

Using MD simulations, Qiao et al. pointed out that the increase in viscosity is significant at the interface of a charged surface and an electrolyte aqueous solution [55], consistent with the viscoelectric theory. On this basis, Hsu et al. investigated viscoelectric effects in nanochannels using a continuum VEDL model and indicated that viscoelectric effects are dominant over other effects such as ionic steric effects and dielectric saturation effects [56]. Due to the presence of the viscoelectric immobile layer, whose length scale becomes comparable to the channel dimension, the electroosmotic mobility can even decrease as surface charge increases, consistent with Qiao et al.'s MD analysis. Both MD and continuum VEDL simulations show that enhancement of viscosity directly suppresses ion diffusivities *D* following the Stokes–Einstein equation:

(20) $$D=\frac{k_{B}T}{6\pi \mu r},$$

where r denotes the ionic radius. In a separate study, Kaji et al. experimentally observed an increase in viscosity and a decrease in DNA diffusivity in nanospace that supports this behavior [57]. Abundant scientific evidence has shown that viscoelectric effects are of crucial importance in various electrokinetic systems, especially at the nanoscale. However, they are largely overlooked in current literature and deserve more attention to elucidate electroosmotic behavior from a fundamental point of view.

Thus far, we discussed the various EDL models that have been developed until now. However, we must note that there is another phenomenon, *electroviscous* effects, which must not be confused with the *viscoelectric* effect. The electroviscous effect is essentially a result of driving the ionic species from one side through a charged micro/nanochannel to the other side using an applied pressure gradient. In this phenomenon, EDL will trap some counter‐ions to drive from the inlet reservoir to the outlet. As a result, we will have nonzero net charge density at the outlet reservoirs, which gives rise to an electric potential difference between the inlet and outlet reservoirs, called *streaming potential* [58, 59, 60]. This nonzero potential difference will generate a backward fluid flow to the pressure‐driven flow as the movement of the ionic species pull the water molecules along with them due to the friction forces between ionic species and water molecules [58, 61, 62]. The net effect of these two driving forces will decrease the flow rate in the pressure‐driven flow direction. This reduction of flow rate due to the resistance of the streaming potential is equal to the increased viscosity of the aqueous solution. For further detail regarding the impact of electroviscous effect on the pressure‐driven flow in micro/nanochannel, refer to Dongqing Li's textbook [58].

Here it is worth pointing out that the distribution of the ionic species at the vicinity of the charged solid surface will be strictly defined by the volume of counter‐ionic species when the solution is concentrated. This phenomenon which is the so‐called Steric effect [63, 64] has been proposed to include the *steric* replusion. Borukhov and Andelman [63] showed that the Steric effect could be incorporated into the Posisson‐Boltzman equation to consider the ionic volume effects which results in ion volume‐dependent counter‐ion concentration. It has been shown that for larger sizes of ionic species, the concentration of counter‐ion species at the vicinity of the charged solid‐liquid interface decreases significantly. The impact of ion size on the electrokinetic flow has been investigated in several previous works [22, 65, 66, 67]. For instance, considering the transport phenomena through nanoporous membranes, the interplay of ultranarrow pore and ionic volume size will determine the electrokinetic transport phenomena [67].

### 2.2 Mechanism of EOF pumping

**Figure 10:**
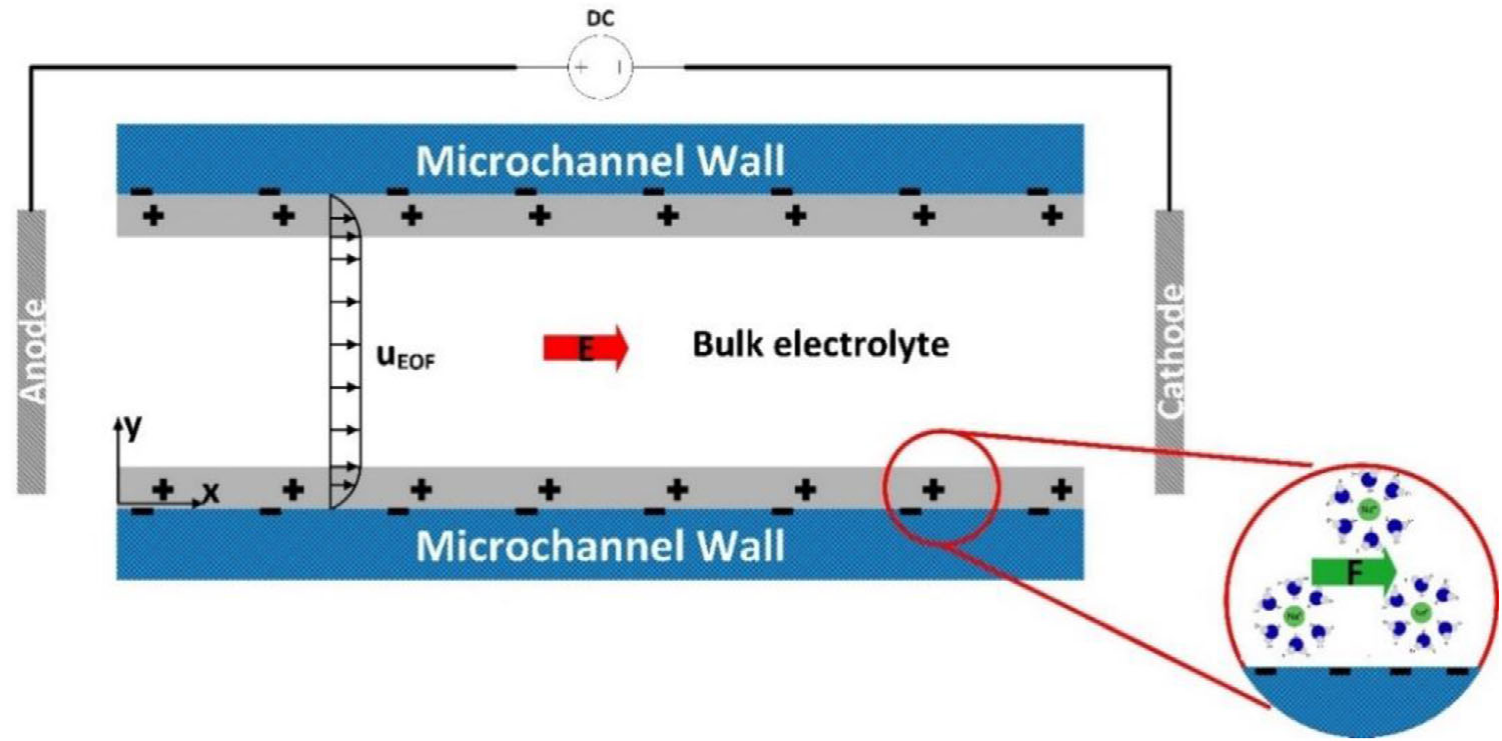
Schematic 2D illustration of EOF in a slit microchannel. The applied electric field (E) drives the counter‐ions in the EDL to the cathode.

In this section, we are going to discuss how the interplay of the applied external electric field and EDL results in pumping of the aqueous solution through the conducting media. Therefore, we focus on a simple flow through two parallel electrically charged plates, called slit microchannel. Figure 10 shows a schematic 2D illustration of the slit microchannel, which is assumed to be negatively charged. EDLs in the vicinity of two walls are shown in gray and are positively charged due to higher counter‐ion concentration.

Recalling what we discussed for Eq. (1), we should point out that the electrical potential at each point in a straight channel without any entrance effect can be considered as a superposition of the applied external electric field and the EDL's electric field. Consequently, we can write the total electric potential as

(21) $$\phi \left(x,y\right)\equiv \phi \;=\;\psi \left(y\right)+\left(\phi_{0}-xE_{x}\right),$$

where $\phi_{0}$ is the anode's potential, x the length from anode, and $E_{x}=\frac{\phi_{0}}{L_{c}}\;$ ($L_{c}\;$denotes the distance between the two electrodes, which can be considered as the microchannel length). If we introduce Eq. (21) into Poisson's equation, the electric potential due to the EDL will remain while the external electric field is a linear function of x.

By applying an external electric field, the whole aqueous solution feels a body force equal to $\rho_{e}\partial \phi /\partial x$. Evidently, the body force can be zero if the concentration of the counter‐ and co‐ions are equal ( $\rho_{e}=\;0$) or nonzero where the local electroneutrality is violated ($\rho_{e}\neq 0$). Therefore, if the zeta potential on the microchannel's walls does not change axially, the Lorentz force because of the external electric field will be the only driving force for the whole solution. Considering $u_{EOF}$ in Fig. 10, the shear force at the middle of the microchannel in EOF can be zero ( $\frac{\partial u}{\partial y}=\;0$). This phenomenon is owing to zero Lorentz in the middle of the microchannel ( $\rho_{e}=\;0$). Later, we will discuss the distribution of the electric potential and velocity inside micro/nanochannels and porous media.

Here it is worth pointing out that although this tutorial focuses on EOF through media composed of nonconductive materials, EOF can still be applicable when the solution is in contact with a conductive medium where the surface is electrically polarized owing to the external electric field, known as induced‐charge electroosmosis (ICEO) [68, 69]. Bazant and Ben [70] theoretically showed that the ICEO can be controlled by a 3D AC applied electric field, enhancing the flow rate of EOF upto 20 times compared to planar AC electroosmosis (AC‐EO) pumps. Regarding this category of EOF, we direct the readers to another review for details [71].

## 3 EOF through microscale pores and channels

In the previous section, we discussed the history and the physics underlying EOF. In this section, we will focus on the broad applications of EOF in the microscale domain, which consists of channels or the porous media. The use of EOF in microfluidic applications is broadening day by day—from lab‐on‐a‐chip devices for biomedical applications to manipulating fluid flows for logical parts of microfluidic chips such as micromixers and microvalves.

### 3.1 EOF through straight microchannels

Following what we discussed in Section 2, if we fabricate a microchannel from materials with functional groups (i.e., – OH) and connect the microchannel to two reservoirs filled with aqueous solutions, upon applying an external electric field through insertion of two electrodes to the reservoirs, the free electric charges in the vicinity of the solid–liquid interfaces (c.f. Section 2.2) start to move toward the counter‐charge electrode. The motion of these ions pulls the solvent molecules due to the frictional forces between molecules.

There are various types of species‐driven methods in lab‐on‐a‐chip devices, namely, pressure‐driven [72], electrokinetic‐driven [73], droplet‐driven [74, 75], and capillary‐driven [76]. Among these species‐driven methods, we will focus on the electrokinetic‐driven method. EOF is one of the categories of species transport methods under the electrokinetic transport phenomena. As we mentioned above, the main advantages of EOF include pumping of solutions without the aid of mechanical parts and easy manipulation (or, in other words, active control) of the fluid flow. In this section, we will try to demonstrate some of these applications and discuss the underlying mechanism.

Considering the structure of lab‐on‐a‐chip devices, which are usually designed to analyze a sample of the analyte [77], one of the key parts of these devices is the micromixer. The rapid mixing of distinct types of species is vital for biochemistry analysis, synthesis of nucleic acids, and even drug delivery. Nguyen and Wu [78] categorized micromixers into passive and active types. The electrokinetic mixing methods are classified as an active mixing method in which the mixing efficiency can be controlled actively through external manipulation. Electrokinetic mixing has attracted the attention of researchers as both pumping and disturbance (which causes the mixing of the species) can be performed simultaneously. It is worth noting that electrokinetic disturbance can be combined with other species‐driven methods, such as pressure‐gradient method, wherein an AC electric field is usually applied perpendicular or alongside the fluid flow. This method will be discussed in detail later.

**Figure 11:**
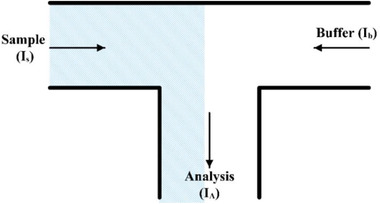
Schematic illustration of the T‐type micromixer with electrokinetically‐driven samples and buffers [79]. Arrows demonstrate the direction of EOF.

As one of the pioneering works in this field, Jacobson et al. [79] demonstrated electrokineticallydriven mixing of species in T‐shaped microfluidic channels. In their experiments, the samples (which must be mixed with buffer solutions) were driven by applying external electric fields to the reservoirs. The external electric field drove both the sample and buffer from separate channels (Fig. 11).

Applying an external electric field at the reservoirs of the sample and buffer initiated their respective flows to the intersection. Consequently, we can consider the ionic current through the analysis channel as a function of the ionic current through the sample (*I_s_*) and buffer (*I_b_*) channels based on the principle of conservation of ionic current:

(22) $$I_{s}+\;I_{b}=I_{A}.$$

In this experiment, the microchannels were designed with the same cross‐sectional areas to ensure that the impact of the cross‐sectional area on the fluid flow of different liquids could be ignored. It is worth pointing out that the applied electric fields on both sample and buffer are the same. Based on these considerations, the ionic current through each channel can be defined as

(23) $$I=\left(WH\right)\;\rho_{e}u,$$

where W, H, $\rho_{e}$, and u denote the width and height of the channel, the net electric charge density, and the fluid flow velocity, respectively. Thus, we can define the sample fraction as

(24) $$n_{s}=\frac{I_{s}}{I_{b}+I_{s}}.$$

The sample fraction could be also obtained based on the channel length *L* using

(25) $$n_{s}=\frac{L_{b}}{L_{s}+L_{b}}.$$

It is noteworthy that the above sample fraction relations are valid when the acquired surface charge on the solid–liquid interfaces could be considered constant. However, this is a simplified assumption that is not realistic in many practical applications, especially when there are asymmetrical bulk properties (i.e., concentration gradient, temperature gradient, etc.).

**Figure 12:**
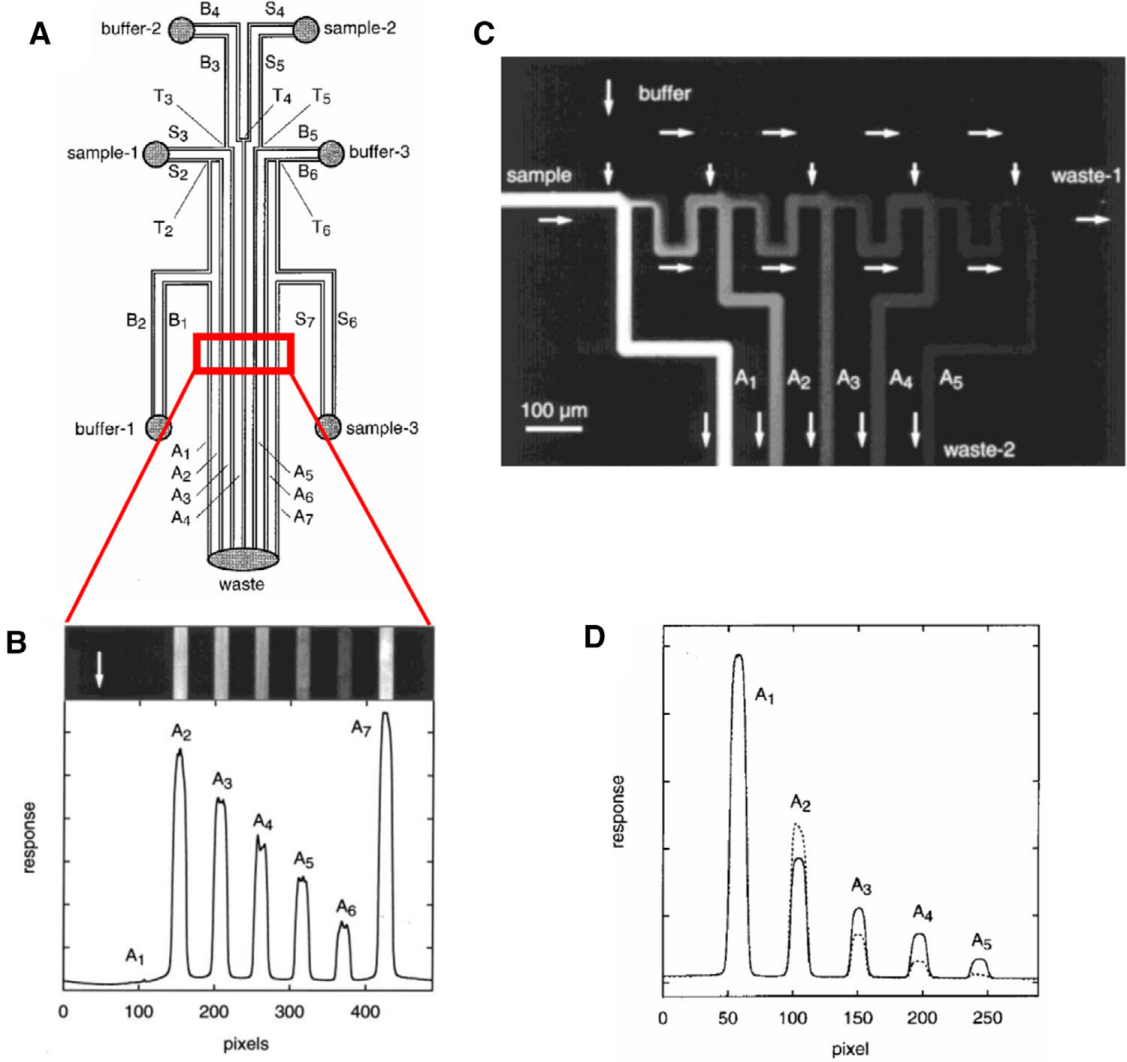
(A) Schematic illustration of the parallel EOF micromixer and (B) the fluorescence image of the mixing results. In this setup, a voltage 1 kV is employed to both buffer and sample reservoirs while the waste reservoir is grounded. (C) Schematic illustration of the serial EOF micromixer with the (D) fluorescence image of the mixing procedure of the buffer and sample. Reprinted with permission from [79]. Copyright (1999) American Chemical Society.

Jacobson et al. [79] proposed microchips for parallel and serial electrokinetic mixing as well (Fig. 12A and B). In the parallel mixing design, several parallel reservoirs for samples and buffers were designed with connected T‐intersections. By applying external electric fields, the fluids from different reservoirs were made to flow and meet at the intersections. To visualize the mixing procedure, a fluorescence solution was added to the sample solutions and the intensity of the fluorescence signal was considered as an index for the mixing capability of the microchip. In parallel mixing, the T‐intersections play a key role. In Fig. 12A, the labels “S,” “B,” and “A” refer to the sample, buffer, and analysis channels. The numerous T‐intersections seen in Fig. 12 enhance the mixing of the species. In serial micromixers, the sample and buffer reservoirs were connected to a serial branch of the microchannels, which increases the meeting intersections of the sample and buffers to increase the mixing efficiency of the species (Fig. 12C and D).

**Figure 13:**
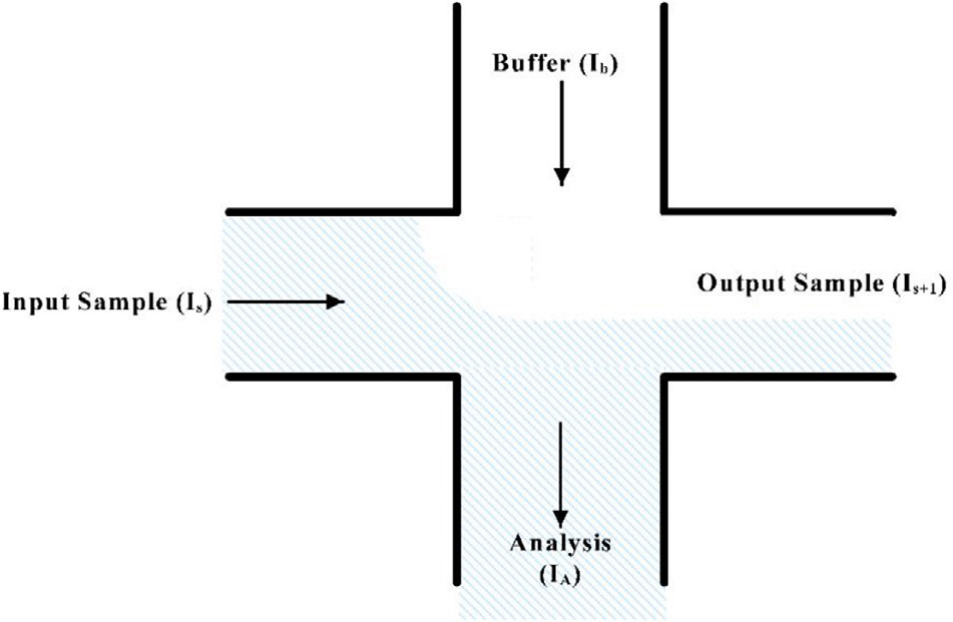
Schematic of mixing at the four‐way intersection of the serial mixing method [79].

In this method, a four‐way intersection plays a key role in enhancing the mixing of the species (Fig. 13). Similar to the parallel mixing method, we can simply derive the conservation of ionic current for the four‐way intersection as

(26) $$I_{s}+I_{B}=I_{A}+I_{s+1}.$$

The above‐mentioned EOF micromixer drives the fluid flow by applying a DC electric field and relies on the geometry of the designed microchips. Although this method is categorized as active micromixing [78], we argue that this should be categorized as “semiactive” micromixing method instead. The main reason in favor of this argument is that the mixing relies enormously on the design of the intersections as well as the number of connected microchannels. However, we must mention that the mixing of the species can still be controlled via an applied DC electric field, which controls the speed of flow of the solutions.

Following the idea that there is a direct relation between perturbation and mixing efficiency, Oddy et al. [80] proposed an interesting design that employed an AC electric field instead of the DC electric field for stronger perturbations in a chamber, and therefore, showed better mixing efficiency. The AC electric field induces oscillating EOF in the microchannels. To determine the oscillating EOF, one may consider the Navier–Stokes (NS) equations under the following assumptions: 1D, low‐Reynolds number flow (consequently ignoring the advective effects on EDL), which can be simplified as

(27) $$\frac{\partial u}{\partial t}=\;\nu \frac{\partial^{2}u}{\partial y^{2}},$$

where u, t, and ydenote EOF velocity, time, and normal direction to the microchannel's walls. The AC electric field affects the solution located in the vicinity of the solid–liquid interface (i.e., EDL). This effect can be introduced in the NS equations by assuming a slip boundary condition as

(28) $$U_{slip}\;\left(y\;=\;0,d\right)=U_{HS}\;\exp\left(i\omega t\right).$$

In this equation, $U_{HS}$ defines the reference velocity for EOF or the Helmholtz–Smoluchowski (HS) velocity [21], which is the result of solving the NS equation for thin EDL (κH≫1, see Eq. (5)) with the nonslip boundary conditions on the microchannel's walls ( $u_{y\;=\;0}=\;0$ and $u_{y\;=\;h}=\;0$). The original steady‐state 1D NS equation in the presence of an applied external electric field is written as

(29) $$0=\nu \frac{\partial^{2}u}{\partial y^{2}}-\rho_{e}\frac{\partial \phi }{\partial x},$$

where the last term on the right‐hand side of Eq. (29) represents the applied external electric body force. As we mentioned in the previous sections, the net electric charge density ($\rho_{e}$) can be obtained by solving the Poisson–Boltzmann equation and recalling the Debye and Hückel assumption:

(30) $$\rho_{e}=-\varepsilon_{0}\varepsilon_{r}\kappa^{2}\psi .$$

By introducing Eq. (8) into Eq. (30), we obtain

(31) $$\rho_{e}=\;-\varepsilon_{0}\varepsilon_{r}\kappa^{2}\zeta \frac{\cosh\left(\kappa y\right)}{\cosh\left(\kappa H\right)}.$$

Then, by introducing Eq. (31) into Eq. (29) and assuming $\partial \phi /\partial x=-E_{x}\;$ (Eq. (21) when $\frac{\partial \psi }{\partial x}=\;0$) we have

(32) $$0=\;\nu \frac{\partial^{2}u}{\partial y^{2}}+\varepsilon_{0}\varepsilon_{r}\kappa^{2}E_{x}\zeta \frac{\cosh\left(\kappa y\right)}{\cosh\left(\kappa H\right)}.$$

An analytical solution to Eq. (32) can be simply derived as

(33) $$u\left(y\right)=-\frac{\varepsilon_{0}\varepsilon_{r}E_{x}}{\mu }\zeta \left[1-\frac{\cosh\left(\kappa y\right)}{\cosh\left(\kappa H\right)}\right]$$

by considering the boundary conditions we mentioned above. Next, let us recall the assumption that the EDL is very thin in comparison with the channel height (κH≫1). As a result, Eq. (33) can be simplified as

(34) $$u\;\left(y\right)=\;-\frac{\varepsilon_{0}\varepsilon_{r}E_{x}}{\mu }\zeta ,$$

and for y=0, we have $u_{y\;=\;0}=U_{HS}\;=\;-\frac{\varepsilon_{0}\varepsilon_{r}E_{x}}{\mu }\zeta$. This is called the HS velocity or the slip velocity at the outer edge of EDL. An analytical solution to Eq. (28) is obtained as [80]

(35) $$u\;\left(y,t\right)=\;f\left(y\right)\exp\left(i\omega t\right).$$

**Figure 14:**
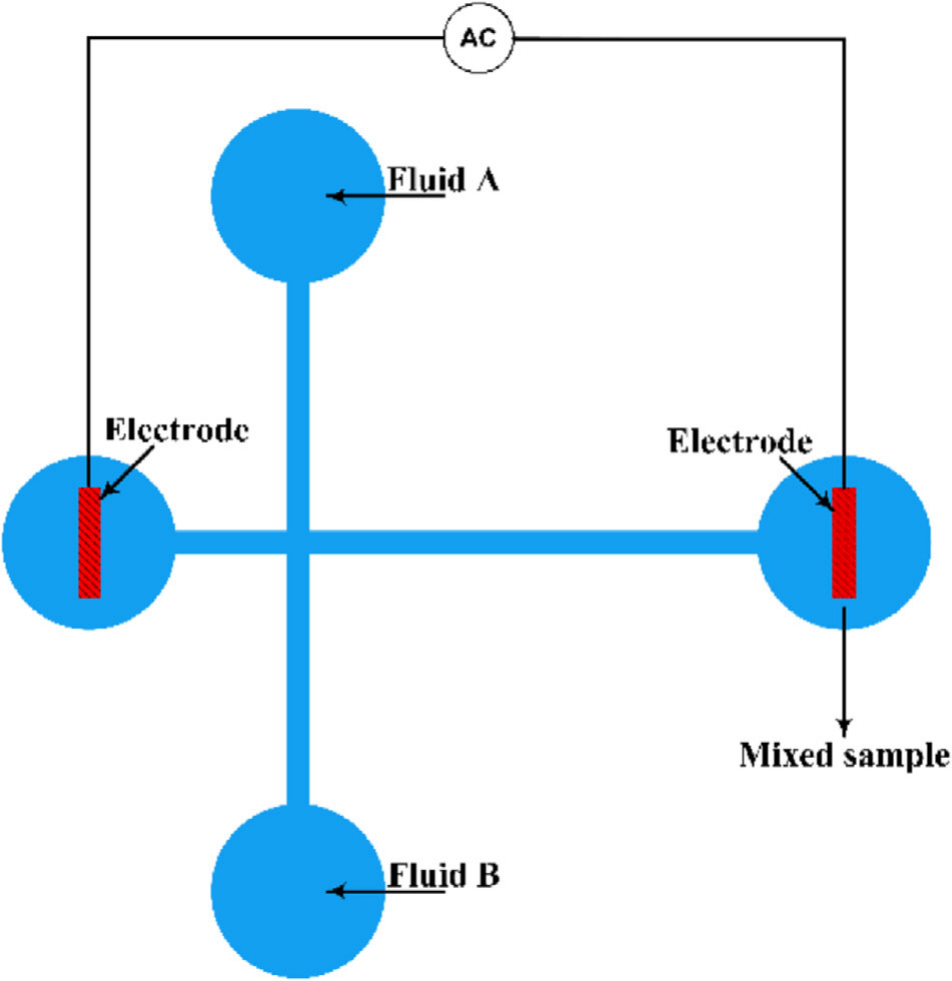
Schematic depiction of the proposed micromixer by Oddy et al. [80]. The two reservoirs on the right‐ and left‐hand side of the microchip are subjected to an AC electric field while samples A and B are pumped into the vertical channel. Applying an AC electric field will disturb the two samples that are entering the straight channel and will enhance the mixing of the species.

**Figure 15:**
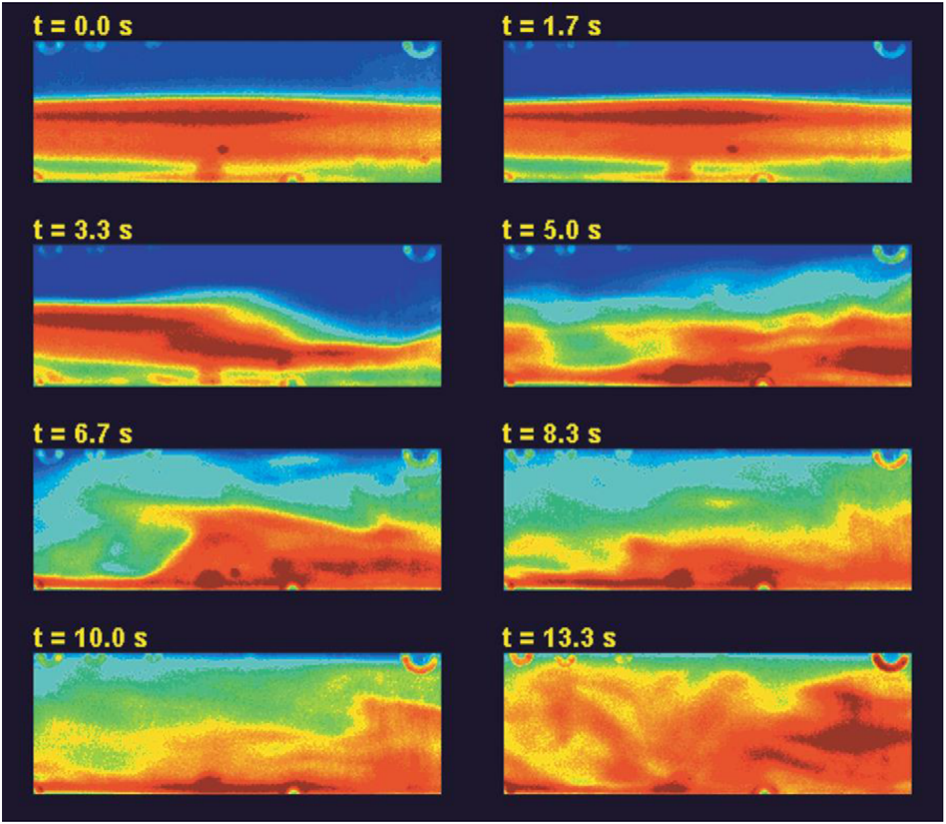
Time‐lapsed frames of the mixing of the species. Starting from a stable interface of the species (*t* = 0.0 s) and its development after the onset of the instability (*t* = 13.3 s). Reprinted with permission from [80]. Copyright (2001) American Chemical Society.

The idea behind the AC‐EOF micromixer is simple and effective. A high‐voltage amplifier is connected to one reservoir of a T‐type microchip made of polydimethylsiloxane (PDMS) and the other end is grounded. The two samples that must be mixed are introduced through the two other reservoirs using a pressure gradient (Fig. 14). The two samples entering the horizontal microchannel will be disturbed due to the applied AC electric field. The oscillation of EOF at the horizontal microchannel will enhance the mixing of the samples (Fig. 15).

**Figure 16:**
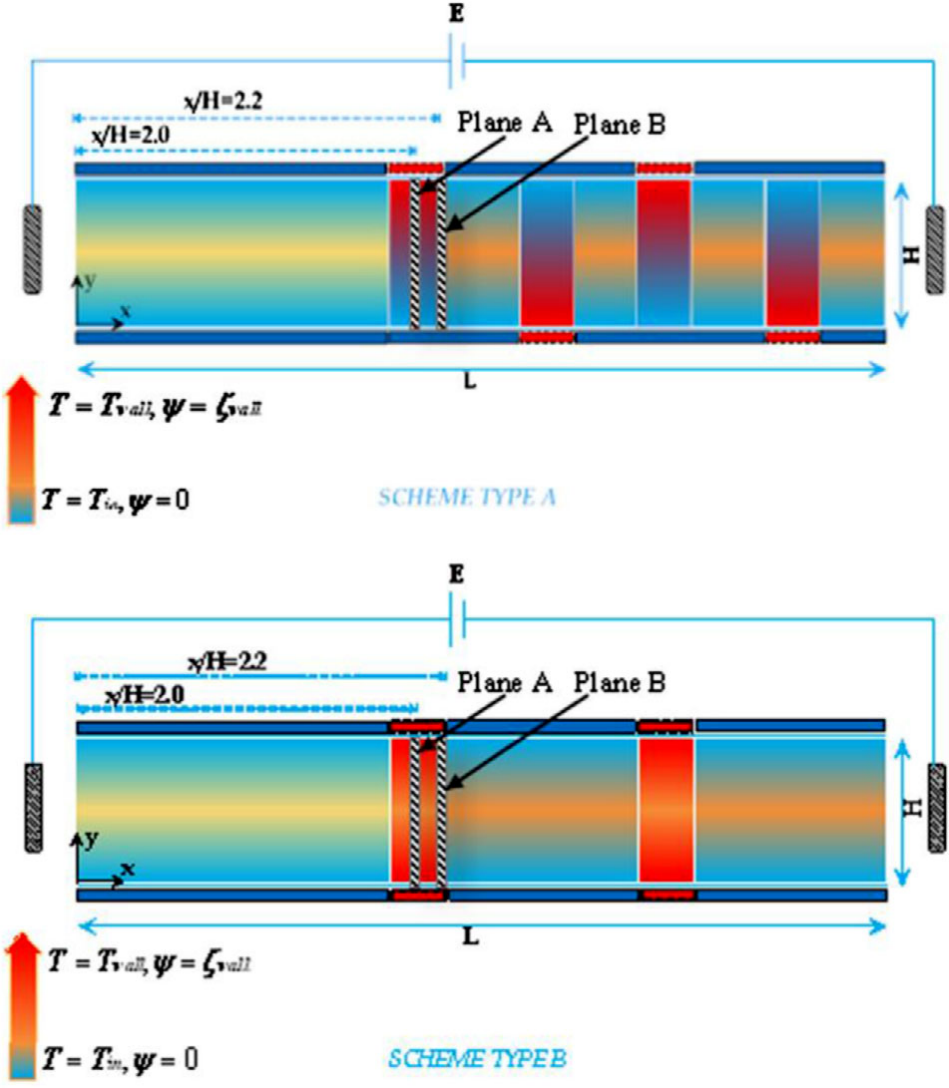
Schematic illustration of micromixers with temperature‐patterned walls. The red blocks on the microchannel walls represent high‐temperature patterns with nonzero surface charge. The other parts of the microchannel were both kept at inlet solution temperature and zero surface charge. The figure has been reprinted from [81].

Thus far, we have shown that the mixing of species could be enhanced by perturbing EOF with the geometrical design or the AC electric field. In addition to these methods, Alizadeh et al. [81] have shown that the mixing of the species could be enhanced by applying temperature‐patterned walls. In this mixing design, patterns of high‐temperature plates were assumed on the microchannel walls in symmetric and asymmetric arrangements (Fig. 16). Patterns with temperatures higher than the inlet solution temperature are shown by red blocks on the microchannel walls. To drive the species at the inlet of the microchannel, different external electric fields were applied along with the same pressure gradient. The modified NS equation for incompressible fluid is

(36) $$\frac{\partial u}{\partial t}+u\cdot \nabla \;\left(u\right)=\;-\nabla p+\nabla \cdot \left(\nu \nabla \left(\rho u\right)\right)+F,$$

where ρ (kg/m^3^) is the density of the electrolyte, p (Pa) is the fluid pressure, ν (m^2^/s) the kinetic viscosity, and F (N/m^3^) is the body force density. It is worth noting that the body force could include all applied body forces such as the electrical body force or the pressure gradients. In this study, the electric body force was defined as $F\;=F_{e}\;+\;F_{p}=\;-\rho_{e}\left(\nabla \varphi +\nabla \psi \right)+\nabla P$, where ∇φ is the external electric field and ∇ψ denotes the internal electric field owing to the distribution of the ionic species.

As the temperature field and the distribution of the electric potential on the microchannel walls are nonuniform, one must solve the Nernst–Planck (NP) equation to obtain the distribution of the ionic and sample species. However, the conventional NP equation is based on isothermal solutions. Therefore, we need to modify it for the nonisothermal scenario. Alizadeh et al. [81] modified the NP equation as

(37) $$\frac{\partial C_{i}}{\partial t}+u\cdot \nabla \;C_{i}=D_{i}\;\nabla^{2}C_{i}+\frac{ez_{i}D_{i}}{k_{B}T}\nabla \cdot \left(C_{i}\nabla \psi \right)-\frac{ez_{i}D_{i}C_{i}}{k_{B}T^{2}}\nabla T\cdot \nabla \psi ,$$

where the last term on the right‐hand side of Eq. (37) is responsible for the temperature gradient effect. This term represents the thermoelectrochemical migration phenomenon, which is a contribution of both temperature gradient and internal electric field. For a detailed discussion regarding this phenomenon and its impact on ionic distribution, see Alizadeh et al. [81].

The two other governing equations that must be solved are the energy equation and the species concentration equations:

(38) $$\frac{\partial T}{\partial t}+u\cdot \nabla T\;=\frac{k}{\rho c_{p}}\;\nabla^{2}T$$

and

(39) $$\frac{\partial C_{s}}{\partial t}+u\cdot \nabla \;C_{s}=D_{s}\;\nabla^{2}C_{s},$$

where T (K) denotes the solution temperature, k the thermal conductivity, and $c_{p}$ is the specific heat capacity. In Eq. (39), $C_{s}$ represents the species concentration and $D_{s}$ is the species diffusion coefficient. For mixing the species in this microchannel, we need to solve the NS, NP, energy, advection‐diffusion, and the Poisson (Eq. (1)) equations in an iterative coupled numerical procedure. The numerical method that Alizadeh et al. [81] used was the Lattice Boltzmann method (LBM), which is a rather novel computational fluid dynamics method that has drawn considerable attention recently [82, 83, 84] owing to its capability and simplicity in modeling multiphysicochemical transport phenomena through structured and unstructured media with micro/nanoscale characteristic length.

**Figure 17:**
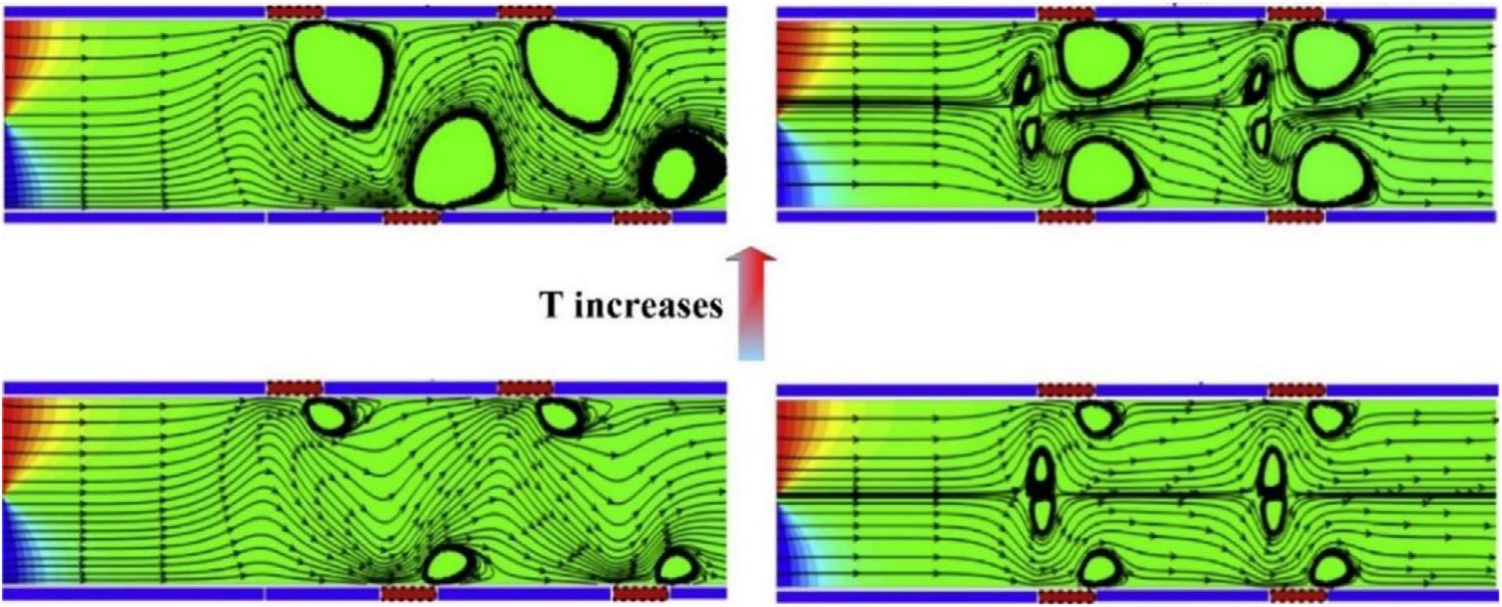
Vortices created due to the temperature‐patterned walls in two arrangements. By increasing the solution temperature, the vortices influence a larger area of the microchannel, which forces mixing of the species. The figure has been reprinted from [81].

The modeling results demonstrated that by increasing the temperature difference between the solution and the patterns on the microchannel's walls, the induced vortices influence a major part of the microchannel. Consequently, the mixing of the species is enhanced (Fig. 17).

Thus far, we have discussed the electrolyte flow through charged media triggered by an external electric field. However, in some cases the electrolyte flow is generated by applying both pressure gradient and external electric field [85, 86, 87, 88]. The governing equations for the combined EOF/pressure‐driven flow are the Poisson equation (Eq. (1)) and the modified NS equation for incompressible flow (Eq. (36)). Equation (36) can be simplified to a 2D system when straight microchannels with the height (*h*) is much smaller than the channel width (*w*). Based upon the aforementioned assumptions, we can derive a modified NS equation as

(40) $$\frac{dp}{dx}=\;\mu \frac{\partial^{2}u}{\partial y^{2}}+\rho_{e}E_{x}.$$

If we introduce the Poisson equation (Eq. (1)) into Eq. (40), then we have

(41) $$\frac{dp}{dx}=\;\mu \frac{\partial^{2}u}{\partial y^{2}}-\varepsilon_{0}\varepsilon_{r}E_{x}\frac{d^{2}\psi }{dy^{2}}.$$

Equation (41) is a linear partial differential equation which justifies the decomposition of the total velocity into the EOF and pressure‐driven velocities as

(42) $$u\;=u_{EOF}\;+u_{p},$$

where $u_{p}$ represents the pressure‐driven fluid velocity. Equation (41) reduces to the HS velocity (Eq. (34)) when there is no applied pressure gradient (i.e., dp/dx= 0). If we nondimensionalize Eq. (41) with $\bar{u}=\;u/u_{HS}$, $\bar{p}=\;p/\left(\frac{\mu u_{HS}}{h}\right)$, $\bar{y}=\;y/h$, $\bar{\psi }=\;\psi /\zeta$, and $\bar{x}=\;x/h$ then we have [85]

(43) $$\frac{d\bar{p}}{d\bar{x}}=\frac{\partial^{2}\bar{u}}{\partial {\bar{y}}^{2}}\;-\frac{d^{2}\bar{\psi }}{d{\bar{y}}^{2}}.$$

Considering the superposition principle for linear partial differential equations, Eq. (43) can be solved as

(44) $$\bar{u}\;\left(\bar{y}\right)=\;-\frac{1}{2}\frac{d\bar{p}}{d\bar{y}}\left(1-{\bar{y}}^{2}\right)+1-\bar{\psi }\left(\bar{y}\right).$$

**Figure 18:**
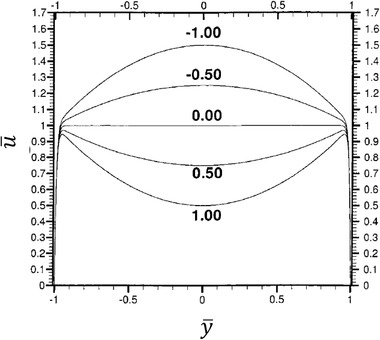
The nondimensionalized combined EOF/pressure‐driven velocity along the cross‐section of the microchannel for different applied pressure gradients. The amounts of $d\bar{p}/d\bar{y}$ curves’ labels. The figure has been reprinted from [85].

Figure 18 shows the nondimensionalized combined EOF/pressure‐driven flow velocity ($\bar{u})$ versus the cross‐section of the microchannel ($\bar{y})$ for different applied pressure gradients. Since the EDL thickness is very thin, for zero pressure gradient (i.e., $d\bar{p}/d\bar{x}$ = 0), it is demonstrated that the flow velocity decays fast by getting far from the microchannel's walls and a uniform plug‐like velocity develops. However, by applying nonzero pressure gradients, the flow velocity departs from plug‐like velocity and which depends on the applied pressure gradient direction.

Electroosmosis can not only drive Newtonian fluids but also non‐Newtonian fluids such as power‐law and viscoelastic [89, 90, 91] fluids. Generally, the non‐Newtonian fluids are defined as fluids in which there is a nonlinear relation between the variation of velocity and the shear stress. EOF of viscoelastic fluid between two parallel plates could be described via the Phan‐Thien‐Tanner (PTT) model [92] which is a model based on the network theory for polymeric fluids. In the viscoelastic EOF, Dhinakaran et al. [91] utilized the Gordon‐Schowalter convected derivative which results in nonzero second normal stress difference in pure shear flow. The impact of EDL and the distribution of the ionic species are considered by solving the Poisson‐Boltzmann equation. The governing equations for the viscoelastic flow could be considered as the continuity and the modified Cauchy equations as [91]

(45) $$\begin{array}{c} \nabla \cdot u=0,\\ \rho \;\frac{Du}{Dt}=-\nabla p+\nabla \cdot \tau +\eta_{s}\nabla^{2}u+F, \end{array}$$

where $\eta_{s}$ is the Newtonian solvent viscosity and τ is the polymeric contribution to the extra‐stress tensor which the solvent viscosity is assumed to be negligible in comparison with the polymeric contribution (i.e., $\eta_{s}=\;0$). The external body force is considered to be obtained by $F\;=\rho_{e}\;E$.

Considering the PTT model to take into account the viscoelastic behavior of the fluid, one has a relation between the extra‐stress tensor and solvent viscosity as [91, 92]

(46) $$f\left(\tau_{kk}\right)\tau +\lambda \;\tilde{\tau }=\;2\eta D,$$

where $D\;=\;(\nabla u^{T}+\nabla u)/2$ is the rate of deformation tensor, λ the relaxation time, η is the polymer viscosity coefficient, and $\tilde{\tau }$ represents the Gordon‐Schowalter convected derivative of the stress tensor defined as

(47) $$\tilde{\tau }=\frac{D\tau }{Dt}\;-\nabla u^{T}\cdot \tau -\tau \cdot \nabla u+\xi \left(\tau \cdot D+D\cdot \tau \right),$$

where ξ denotes the slip between the molecular network and the continuum medium [92]. Dhinakaran et al. [91] proposed an analytical solution for the set of governing equations. Their solution showed that the normal and shear stresses are approximately zero at the centerline of the channel while they rapidly rise by approaching toward the channel's walls.

### 3.2 EOF through microporous media

In the last section, we discussed the solution transport through the electrically charged microchannels with the application in micromixers as an essential part of the lab‐on‐a‐chip devices. In this section, we will study the applicability of EOF through complex electrically charged domains such as porous media. Porous media can be defined as any naturally (i.e., underground soil or brain) or artificially (i.e., filtration or fuel cell membranes) produced complex material with a very high surface‐to‐volume ratio. Applications of such porous media are very broad—from biology [93, 94] and environmental science [25, 26, 95, 96, 97, 98] to geology [99, 100, 101, 102, 103]. This section aims to highlight EOF through microporous media by focusing on relevant applications. Here, we must point out that microporous media are characterized as porous media with an average void size on the order of a few micrometers.

**Figure 19:**
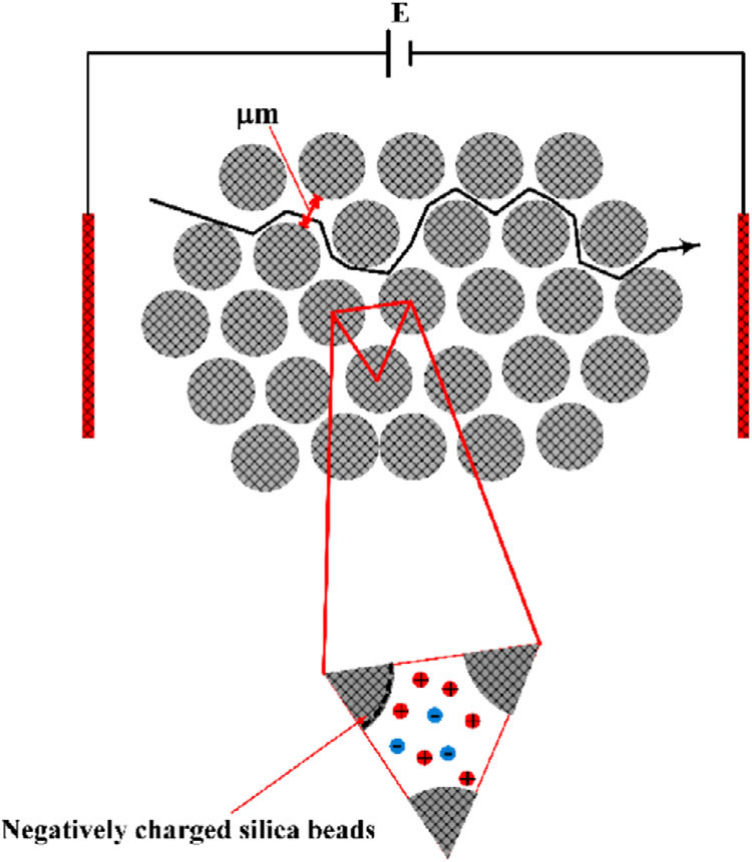
A schematic illustration of micron‐sized silica beads. The silica beads will acquire a negative surface charge due to the chemical reaction with the solution (c.f. Section 2.1). EOF is generated by applying an external electric field to both ends of the microporous media. The negatively charged silica beads will generate a polarized layer of the solution (EDL) and the external electric field will push the solution in the vicinity of the solid surface.

Historically, Paul et al. [104] reported that the electrokinetic phenomena could induce high pressures that are suitable for pumping liquids through microporous media. The experiments were performed for a pack of micron‐size silica beads (Fig. 19). The difference in the work done by Paul et al. [104] with what we mentioned in Section 2 is in the capability of EOF in generating high pressures through porous structures. Paul et al. have shown that EOF could generate pressures as high as 55 MPa. To consider the porous media, they assumed that a porous medium is a composition of microcapillaries. The characteristic properties of any porous media could be defined by several parameters such as porosity (φ) and tortuosity (τ). Porosity is defined as the volume fraction of the voids and tortuosity is simply defined as the length of the tubules per unit length of the medium. A tortuous porous medium will reduce EOF velocity. Consequently, the averaged EOF velocity in a porous medium for a given porosity (φ) and tortuosity (τ) is [104]

(48) $$U_{0}=\;-U_{HS}\left(1-f\left(\frac{\kappa d}{2},\zeta \right)\right)\frac{\varphi }{\tau^{2}},$$

where d denotes the effective pore diameter for the packed beads (which has a direct relation with the beads’ diameter) and f is a function that is responsible for any overlapping effect of EDL. Clearly, for the nonoverlapped regimes, the average EOF is the HS velocity. They proposed that for a very thin EDL, the flow rate must be an independent function of the pore diameter and is proportional to the applied external electric field (E=V/L).

If we assume that porous media is a combination of many capillaries, then the averaged EOF velocity combined with the applied pressure gradient could be obtained as [104]

(49) $$U_{m}=\;-\frac{\nabla Pd^{2}}{8\eta }-U_{HS}\left(1-f\left(\frac{\kappa d}{2},\zeta \right)\right).$$

To obtain the pressure gradient due to the applied external electric field, we need to assume a zero net flow, which means EOF is opposed to a pressure‐driven flow. Therefore, we have

(50) $$\Delta \;P_{EOF}=\;-\frac{32\varepsilon_{0}\varepsilon_{r}\zeta }{d^{2}}\left(1-f\left(\frac{\kappa d}{2},\zeta \right)\right)\Delta V.$$

Equation (50) indicates that the induced pressure gradient owing to the applied external electric field is solely a function of the effective pore diameter and the electrochemical properties of the porous medium.

**Figure 20:**
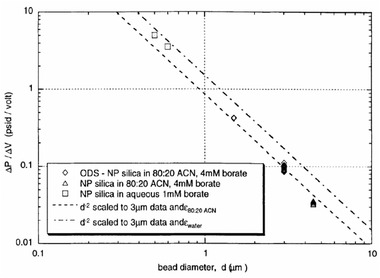
Pressure generated by employing an external electric field toward a micro‐size packed silica beads. The experimental measurements demonstrate the generated pressure gradient divided by the applied external electric field versus the bead diameter. The figure has been reprinted from [104].

Paul et al. conducted simple experimental measurements to obtain the induced pressure due to the electroosmotic pumping effect. In their setup, fused silica capillaries were packed with nonporous silica (NPS). The porosity of their medium was 0.32. The packed silica beads were placed in between two fluid reservoirs, which were filled with a mixture of 80:20 of acetonitrile and water buffered with 4 mM aqueous sodium tetraborate and pure water with 1 mM borate. To make sure that the EDLs in the pores of the silica beads pack are not on the overlapped regime, we can simply calculate the EDL thickness by employing its definition $\kappa \;=\sqrt{\left(\frac{2e^{2}c_{i}^{b}}{\varepsilon_{0}\varepsilon_{r}k_{B}T}\right)}\;,$ which results in 5 to 6 nm. As we mentioned above, the measured porosity of the pack was 0.32, which suggests that the average pore diameter to the Debye length would be 10. This indicates that EDLs were not generally overlapped. The experimental setup consisted a packed capillary with one end open to the ambient pressure while a platinum electrode was introduced to the reservoir. The other end of the packed capillary was fitted into a plastic HPLC “T” junction which the side leg was fitted with a platinum wire electrode. Clearly, the external electric field will be applied to both ends of the packed capillary through the designated electrodes. The remaining third leg of the “T” was designed to attach to different diagnostics. Paul et al. [104] measured the pressure generated through EOF by measuring the compression length of an air gap that is trapped in a long capillary tube that is sealed on one end. Figure 20 depicts the experimental measurements for different micro‐sized silica beads and the generated pressure gradient for different bead diameters. In this figure, ODS represents OctaDecyl Silyl (C_18_H_37_Si(CH_3_)_2_), which is a hydrocarbon group with a cation exchanger C_18_H_37_Si. The dashed lines represent the predicted values of $\Delta P_{EOF}/\Delta V$ by Eq. (50) which are scaled to the mean of the 3 μm ODS coated NPS beads.

As we know, the acquired or applied surface charge at the solid–liquid interface plays a key role in EOF. Therefore, it is of utmost importance to study the influence of surface charge (σ) or zeta potential (ζ) on the ionic species’ transport through the microporous media. Historically, researchers have attempted to (I) prescribe the surface charge and zeta potential as an independent parameter, namely homogeneous surface charge, or (II) obtain the local surface charge by solving the EDL models (i.e., Section 2.1), namely inhomogeneous surface charge. Clearly, the first method brings simplicity to the complex electrokinetic transport phenomena through complex geometry. However, these results may neglect the possibly significant impact of the local surface charge on the species’ transport properties.

For homogeneous surface charge, there is a large body of literature that focuses on the theory [105, 106, 107, 108, 109, 110] and experiment [111, 112] of EOF through porous media. The common assumption among the early works is that the EDL is very thin compared with the characteristic pore size. Therefore, we can introduce a slip velocity (the HS velocity) at the solid–liquid interface, which includes the impact of the EDL on the solution.

Starting from a pioneering work, Coelho et al. [113] briefly reviewed the previous attempts until 1996 and they attempted to study the impact of EDL thickness on EOF through porous media with an intermediate range of double layer thickness. Coelho et al. attempted to, first, propose the general electrokinetic equations that govern the transport of the ionic species and the solution through an infinite spatially periodic porous medium. Subsequently, the proposed equations were linearized by introducing ionic potentials as proposed by O'Brian [112, 114]. Finally, they proposed the general numerical simulation results and compared the simple cases with the available analytical solutions.

The general electrokinetic equations for porous media are the same as those proposed for the straight channel (see Section 3.1). The flux of the ionic species is

(51) $$J_{i}=\;-D_{i}\nabla C_{i}-\frac{ez_{i}D_{i}C_{i}}{k_{B}T}\nabla \psi +C_{i}u,$$

a superposition of the diffusion (the first term on the right‐hand side), electromigration (second term), and convection (third term). Equation (51) is obtained by applying the continuity equation,

(52) $$\frac{\partial C_{i}}{\partial t}+\nabla \cdot \;J_{i}=\;0.$$

Regarding the fluid flow, we can simplify the NS equation because fluid flow through compact porous media inherently has a very low Reynold's number. This low Reynold's number justifies the utilization of the Stokes equations with transient specification as [113]

(53) $$\mu \nabla^{2}u-\rho_{e}\;\frac{\partial u}{\partial t}=\;\nabla p+F,$$

where μ represents the dynamic viscosity, p is the pressure, and F is the Lorentz electric body force (which we have defined in Eq. (36)). Essentially, we need to solve Poisson's equation (Eq. (1)) to obtain the distribution of the electric potential owing to EDL and the ionic species as charged particles. To solve the governing equations, we need to define proper boundary conditions. The boundary conditions for porous media in which the solid–liquid interfaces were assumed to be impenetrable are the same as those we introduced for the straight channels. Hence, we have

(54) $$\begin{array}{c} n\cdot \;J_{i}=0,\\ u_{s}=0,\\ \psi =\zeta , \end{array}$$

where n denotes the normal unit vector to the solid–liquid interface and $u_{s}$ is the fluid flow velocity on the solid–liquid interface. The first boundary condition represents the zero ionic fluxes into the solid surface. It is assumed that electric potential ψ on the interface is the Dirichlet type. Here, we should note that the boundary condition for Poisson equation could be considered as Neumann‐type if we set the surface charge on the solid–liquid interface instead of the electric potential. The relation between the surface charge and the electric potential on the solid–liquid interface could be obtained by considering the definition of balancing the surface charge with the free net electric charge density in the solution:

(55) $$\sigma_{s}=\;-\int_{0}^{\infty }\rho_{e}dy.$$

If we introduce Poisson equation into Eq. (55), then we have

(56) $$\sigma_{s}=\varepsilon_{0}\;\varepsilon_{r}{\left[\frac{d\psi }{dy}\right]}_{0}^{\infty },$$

where at infinity or in symmetrical boundary conditions, we have $\frac{d\psi }{dy}=\;0$. Finally, we obtain

the more general form of Eq. (57), which is given as

(57) $$\sigma_{s}=-\varepsilon_{0}\varepsilon_{r}\frac{d\psi }{dy}{|}_{0}.$$

(58) $$\sigma_{s}=-\varepsilon_{0}\varepsilon_{r}\nabla \psi \cdot n.$$

Consequently, for Poisson equation boundary conditions, we can also prescribe the surface charge and obtain the electric potential with Neumann‐type boundary condition.

**Figure 21:**
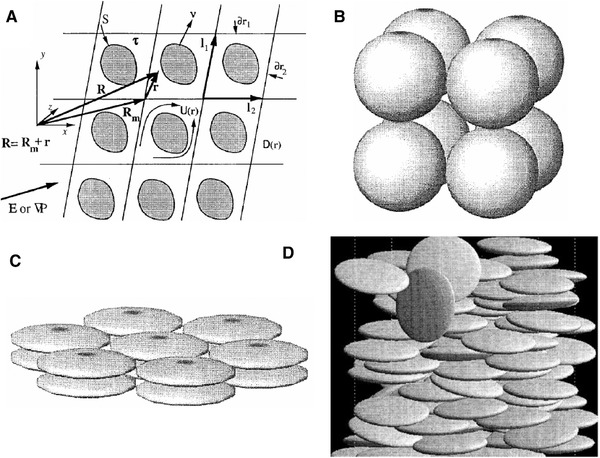
Schematic illustrations of (A) periodic medium, (B) simple cubic array of spheres, (C) orthorhombic lattice, and (D) a bed of ellipsoids obtained via sequential deposition. All the structures have been reprinted from [113].

To complete the problem formulation, we need to determine the structure of the porous media. In this regard, Coelho et al. [113] considered several ordered and disordered microstructures. The ordered microstructures could be considered as the periodic media in which the locations of the grains and, consequently, the pore domain could be described easily by simple vectors. It is worth noting that the diameter of the grains must be the same. As Fig. 21A shows, the 3D space of the ordered porous media could be defined in $R^{3}$ as

(59) $$R\;=\;r+\;R_{m}=\;r+\left[R\right]\cdot \left[n\right],$$

where $\left[R\right]=\;\left[I_{1},I_{2},I_{3}\right]$ and $\left[n\right]=\;\left[n_{1},n_{2},n_{3}\right]$ are the three basic vectors that characterize the unit cell of the ordered porous medium and the trio of integers that belong to $Z^{3}$, respectively.

Coelho et al. linearized the governing equations by assuming that the deviation of the system from equilibrium could be very small when the applied external electric field is small compared to the EDL electric field. As a result, the concentration of the ionic species, electric potential, pressure gradient, and the velocity could be written as

(60) $$\begin{array}{c} C_{i}=C_{i}^{0}\left(r\right)+\delta C_{i}\left(R,t\right),\\ \psi =\psi^{0}\;\left(r\right)+\delta \psi \left(R,t\right),\\ p=p^{0}\;\left(r\right)+\delta p\left(R,t\right),\\ u=\delta u\left(r,t\right), \end{array}$$

where the superscript “0” denotes the amount of parameter in the equilibrium state. It is worth noting that the fluid flow velocity in equilibrium state must be equal to zero, which represents the state when there is no applied external body force. Coelho et al. [113] introduced the above definition of parameters into the governing equations and solved them numerically. For further details regarding the linearized dimensionless governing equation, refer to their work. The most interesting results from their numerical simulation is in the limit of the thin EDL when the slip velocity at the edge of EDL ($U_{HS}$) is a valid assumption. The velocity in the bulk region of the pore (far from the solid–liquid interface where the electric potential due to the walls would be near zero) is a function of the local electric field. This implies that the fluid flow in the pore space of the porous media could be considered as an inviscid flow (a flow in which the viscosity or the shear stresses are equal to zero) and is governed by Laplace's equation. However, for the thicker EDL, they showed that the whole pore space contributes to the drag force on the porous media wall owing to the fluid viscosity. Therefore, it is reasonable to make an analogy between the pressure‐driven flow and an EOF with thicker EDL which the major solution in pore will be driven by the applied electric field because of nonzero net electric charge density. We will discuss in detail the contribution of comparable EDL thickness with the characteristic length of the channel or pore in the following sections.

Later, Gupta et al. [115] attempted to model the electroosmosis through solid porous media by considering a high zeta potential. The high zeta potential will not allow us to linearize the right‐hand side of the Poisson–Boltzmann equation (Eq. (3)). As a result, Gupta et al. attempted to solve the governing equations numerically for different types of porous media.

**Figure 22:**
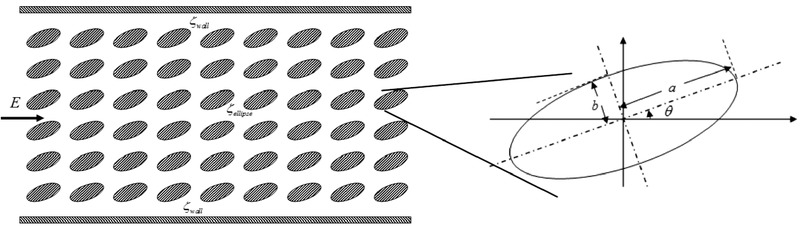
The schematic illustration of the porous medium which is a pack of ellipses in a microchannel. The height of the microchannel is 1 μm. The figure has been reprinted from [110].

In another study, Wang et al. [110] investigated EOF in anisotropic porous media, that is, porous media with inhomogeneous directionality of grains. The porous medium in the study was created by considering arrays of ellipses which were packed in a microchannel with 1 μm height. It was assumed that the microchannel walls and the solid–liquid interface of the ellipse grains with electrolyte solution were charged equally, that is, $\zeta_{wall}=\zeta_{ellipse}=-50$ mV. The bulk concentration of the ionic species was selected to be $n_{b}=10^{-4}\;$ M. In order to initiate EOF through the microporous medium, an external electric field with strength of E=5 KV/m was applied. As Fig. 22 shows, we can make the porous medium isotropic or anisotropic by changing the orientation angle θ. Wang et al. [110] simply picked a and b in such a way to have a porous medium with pore sizes 0.7 μm.

**Figure 23:**
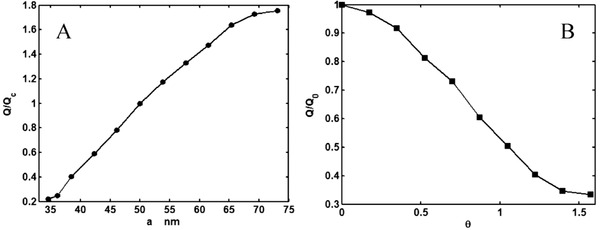
Normalized EOF rate versus (A) semimajor axis and (B) the orientation angle. For (A), the orientation angle is considered to be θ= 0 and for (B) it is assumed that a= 61.5 nm and b= 40.6 nm. The EOF rate was normalized by the flow rate when θ= 0. The figures have been reprinted from [110].

They solved the NS equation combined with the Poisson equation where the distribution of the ionic species was obtained by employing the Boltzmann distribution [110]. The modeling result demonstrated that the EOF rate is a function of the orientation angle and the size of the ellipse's semimajor axis. It has been shown that by increasing the ellipse's semimajor axis, the EOF rate increases. This means that if we decrease the pore size or increase the porosity of the porous medium in the *y* direction, the volumetric flow rate of the solution will increase (Fig. 23A). This behavior of the EOF rate as a function of the semimajor axis of a pack of ellipses is expected when the orientation angle is assumed to be θ= 0 because the packs of ellipses will behave as several parallel microchannels. Thus, more electrolytes will flow in the external electric field direction and less will flow normal to the electric field. In contrast, we expect that by increasing the orientation angle (θ), the EOF rate decreases. Figure 23B shows the impact of the orientation angle on the EOF rate. It is shown that by increasing the orientation angle, the EOF rate will decrease.

**Figure 24:**
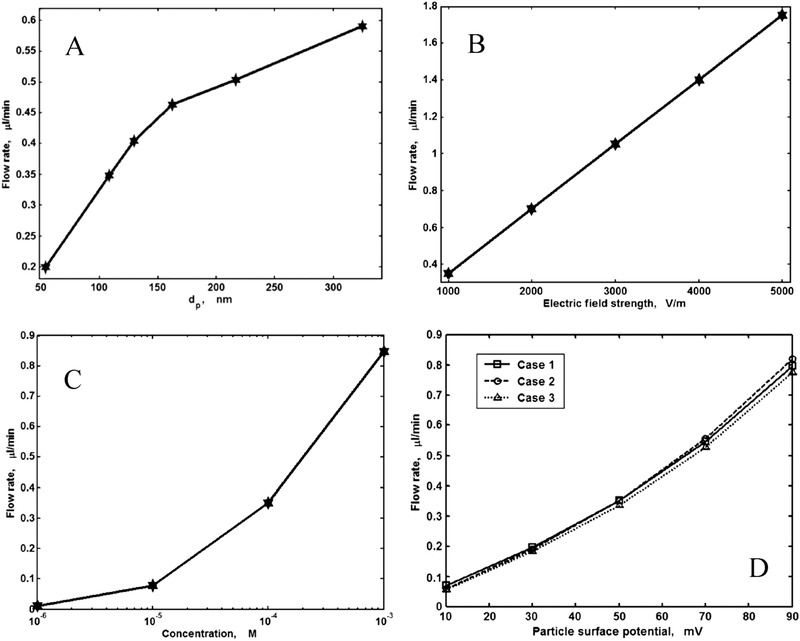
EOF rate versus (A) particle size, (B) applied external electric field strength, (C) bulk ionic concentration, and (D) the particle zeta potential. The figures has been reprinted from [116].

**Figure 25:**
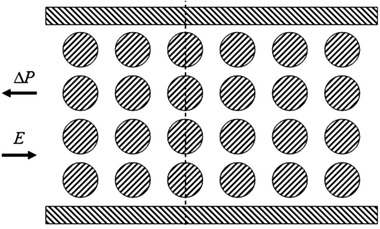
Microporous medium, which is a pack of spherical particles with a structured distribution. The walls of the microchannel were charged as $\zeta_{w}$ = −50 mV while the zeta potential of the particles could be changed as a parameter to study the EOF rate. The external electric field (E) and the generated pressure gradient (ΔP) are shown in this schematic illustration. The figure has been reprinted from [116].

Prior to this work, Wang et al. [116] studied the EOF pumping effect in microporous media by considering the particle size effects (Fig. 24A), external electric field (Fig. 24B), bulk ion concentration (Fig. 24C), and the prescribed zeta potential on the particles (Fig. 24D). The microchannel had a 1 μm height and the structured porous medium was considered to be a pack of spheres (Fig. 25). The modeling results revealed that by increasing the particle size, the EOF rate increased significantly. In this structure, it was assumed that the particle sizes are changing while the porosity is constant. This situation results in a different particle number when we change the particle size.

As a step forward in understanding EOF through microporous media, Wang and Chen [117] utilized a random generation‐growth method for reproducing the structure of the porous media with prescribed porosity and grain sizes [118, 119]. It was assumed that the charge of the solid–liquid interface is homogeneous and was prescribed as an input boundary condition for Poisson's equation. The governing equations are the same as those we mentioned previously (Eq. (1), (52), and (53)). They utilized LBM to solve the governing equations (Section 3.1).

**Figure 26:**
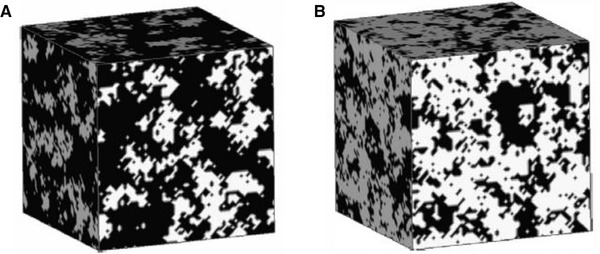
Generated microporous structure using the random generated‐growth method for a 60×60×60 grid system. (A) represents the porous medium structure with porosity 0.3 and (B) porosity 0.6. The structures have been reprinted from [117].

The 3D randomly generated microporous media (Fig. 26) were subjected to an external electric field (E) and EOF initiated through the negatively charged micropores. Their modeling results showed that EOF permeability $\kappa_{e}=\bar{u}\;/E$ (m^2^/sV), which is defined as the averaged velocity over the strength of the applied external electric field, has a nonlinear relationship with porosity.

**Figure 27:**
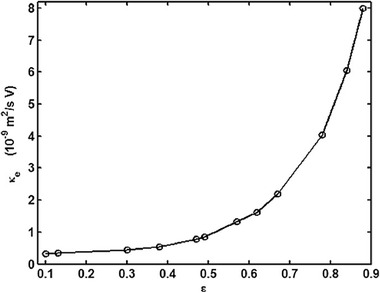
Electroosmotic permeability ($\kappa_{e}$) versus porosity (ε) of a porous medium for the reservoir concentration $n_{b}=10^{-4}\;$ M, ζ=−50 mV, and $E\;=10^{4}\;$V/m. The figure has been reprinted from [117].

As Fig. 27 demonstrates, by increasing the porosity of the porous medium for ε<0.5, the electroosmosis permeability increases slightly. However, for porosities beyond 0.5, the electroosmotic permeability increases dramatically with the porosity. This behavior of porous media was shown by Wang et al. [120] to be due to the numerical instability of the lattice Boltzmann scheme they selected for 3D modeling of complex geometry.

**Figure 28:**
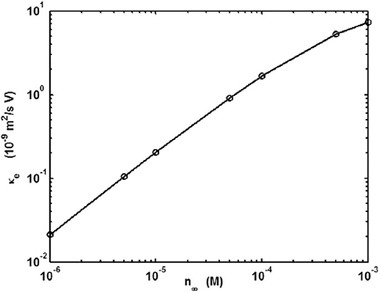
Electroosmotic permeability as a function of bulk ion concentration. The modeling results have been reprinted from [117].

In addition, Wang and Chen [117] investigated the influence of bulk ion concentration and prescribed zeta potential on the EOF permeability (Fig. 28).The modeling results are significant, in that they demonstrate the importance of electrochemical properties of the solution and the solid–liquid interface in determining EOF through microstructure porous media.

Regarding the impact of bulk ionic concentration, modeling results of Fig. 28 demonstrate that electroosmotic permeability always increases with bulk ion concentration. However, this increase in electroosmotic permeability slows down for higher bulk ion concentrations.

We have mentioned (Section 2.1) that the zeta potential and the surface charge of chemically active solids are a function of the bulk ion concentration and the solution pH. Thus, it is of utmost importance to determine the solid–liquid interface electric potential based on the local solution properties. Following Wang and Chen's [117] study, Wang et al. [120] attempted to study multiphysicochemical transport through randomly generated porous media by considering the zeta potential as a result of the local solution properties. For the electric boundary conditions on the solid–liquid interface, they utilized the BS model, which is based on the dissociation of the silanol groups:

(61) $$\mathrm{SiOH}⇌\mathrm{Si}O^{-}+H^{+}.$$

**Figure 29:**
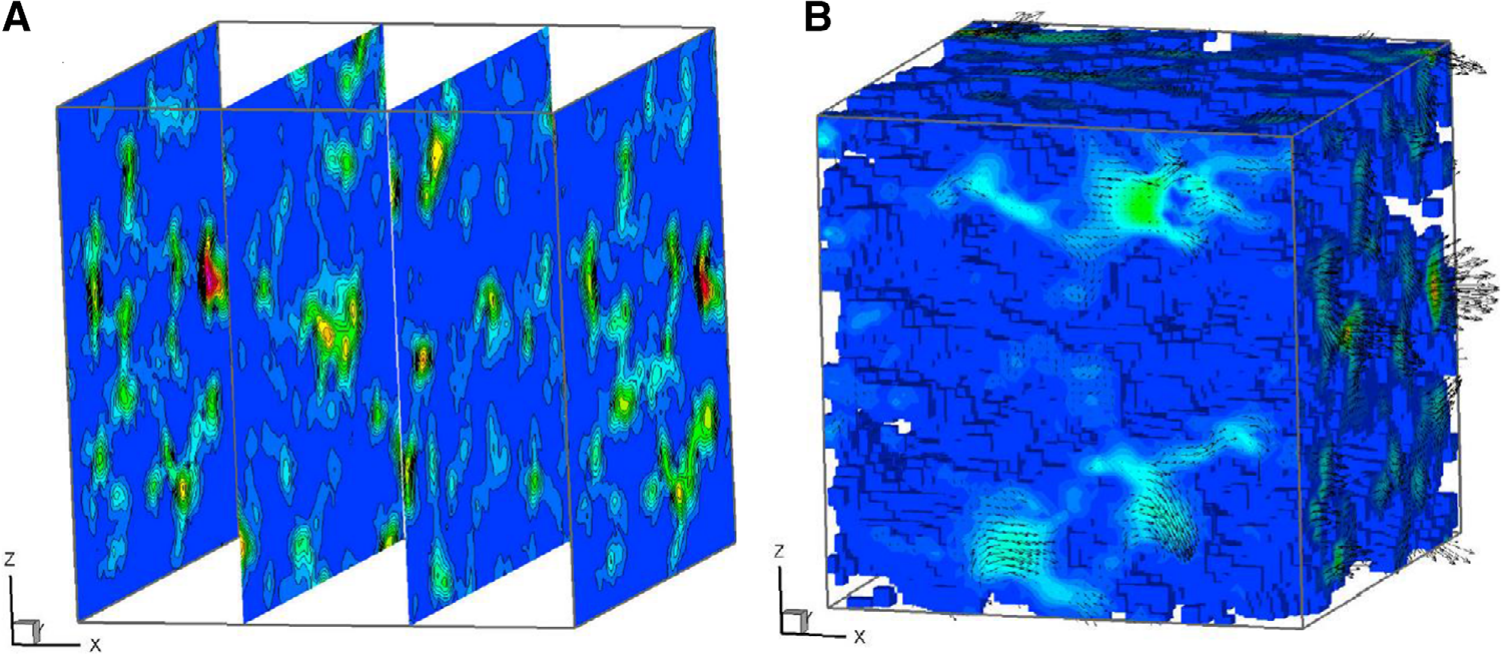
Electric potential and EOF vectors for a randomly generated porous media with porosity ε=0.14. (A) Four slices of the porous media from inlet to the outlet at x=0, 1/3, 2/3, and 1. (B) The velocity vector field and the contour of EOF *x* direction velocity. The figures have been reprinted from [120].

Behrens and Grier [121] proposed that zeta potential could be expressed as a function of surface charge density (σ) and defined as

(62) $$\zeta \;\left(\sigma \right)=\frac{kT}{e}\;\ln\left(-\frac{\sigma }{e\Gamma +\sigma }\right)-\left(pH-pK\right)\frac{kT\ln\left(10\right)}{e}-\frac{\sigma }{C},$$

(63) $$\sigma \;\left(\zeta \right)=\frac{2\varepsilon_{r}\varepsilon_{0}kT\kappa }{e}\;\sinh\left(\frac{e\zeta }{2kT}\right),$$

where Γ=8, pK=7.5, and C=2.9 denote the surface density of chargeable sites on the silica surface (nm^−2^), the dissociation equilibrium constant, and the Stern layer phenomenological capacity (F/m^2^). Clearly, the two Eqs. (62) and (63) are a set of nonlinear equations that must be solved numerically, combined with the Poisson–Boltzmann and NS equations. Wang et al. [120] solved the governing equations by using the 3D LBM method. Their modeling results demonstrate the distribution of the electric potential that is obtained by solving Eq. (62) (Fig. 29A) and EOF velocity vectors (Fig. 29B).

**Figure 30:**
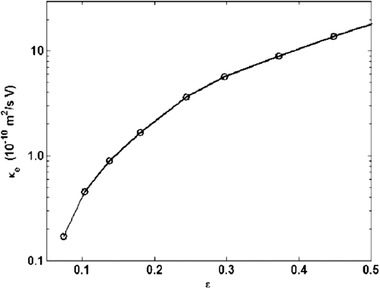
EOF permeability versus porous medium porosity (ε) for solution with $n_{b}=\;1\times 10^{-5}$ M and ζ=−50 mV. The figure has been reprinted from [120].

This work could be distinguished from the previous one (Wang and Chen [117]) as it highlighted the fact that the zeta potential cannot be changed as an independent input parameter according to bulk ion concentration and solution pH. Consequently, if we need to study the impact of zeta potential on electroosmotic permeability, we need to change the pH or the bulk ion concentration. In their work, Wang et al. [120] showed that EOF permeability sharply changes as a function of the porosity even for ε<0.3 (Fig. 30). As we mentioned previously, these results are in clear contradiction with their previous numerical prediction (Wang and Chen [117], Fig. 28). These contradictory results were interpreted to be due to the numerical instability of the former study.

**Figure 31:**
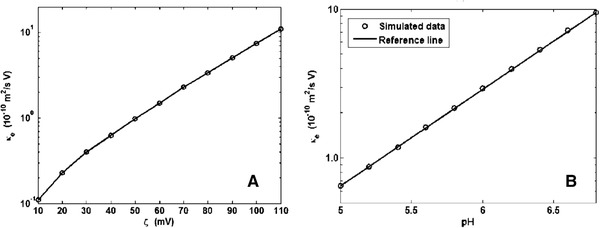
Impact of (A) zeta potential when the bulk ion concentration is $n_{b}=\;1\times 10^{-5}$ M and T=293 K and (B) solution pH on electroosmotic permeability when ε=0.14 and $n_{b}=\;1\times 10^{-5}$ M. The figures have been reprinted from [120].

Wang et al. [120] studied the effects of zeta potential (Fig. 31A) or, consequently, solution pH (Fig. 31B) on the electroosmotic permeability. Interestingly, the modeling results indicated that there is no electroosmotic permeability saturation for high zeta potentials. This finding was similar to that of their previous work (Wang and Chen [117]) (Fig. 29B). They showed that even for zeta potentials higher than 100 mV, the logarithm of electroosmotic permeability could be considered to be increasing approximately linearly. They concluded that these results, which are in contradiction with their previous study, could be due to numerical instability and not a physical effect. This increase in zeta potential for a fixed bulk ion concentration is due to the increase in the solution pH. In Fig. 31B, the electroosmotic permeability is demonstrated as function of pH.

**Figure 32:**
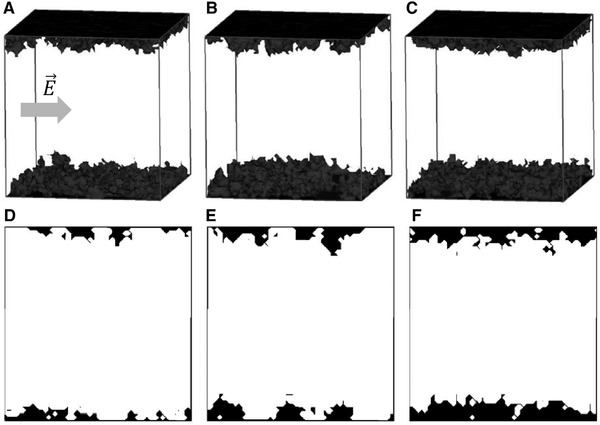
Three randomly generated roughness on the microchannel walls with (A) $s_{d}$ = 0.03 and $V_{R}$ = 0.06; (B) $s_{d}$ = 0.01 and $V_{R}$ = 0.06; (C) $s_{d}$ = 0.03 and $V_{R}$ = 0.01, where $s_{d}$ denotes the roughness distribution probability and $V_{R}$ denotes the total volume fraction of roughness. The *x*–*z* cross‐section of the microchannel with roughness is shown as (D) to (F). Reprinted with permission from [122]. Copyright (2009) American Chemical Society.

**Figure 33:**
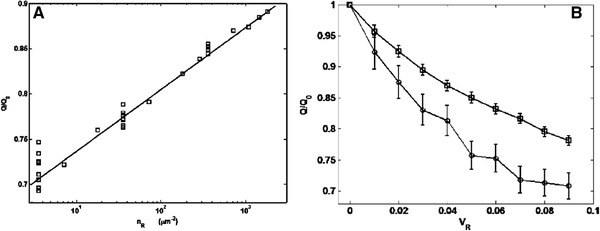
Normalized EOF rate versus (A) roughness number density with $V_{R}$ = 0.05 and λ/H = 0.1683 and (B) total roughness volume fraction, where the squares are modeling results for $n_{R}$ = 360/μm^2^ and the circles are $n_{R}$ = 36/μm^2^. Reprinted with permission from [122]. Copyright (2009) American Chemical Society.

As a combination of EOF through a straight microchannel and a porous medium, EOF through a microchannel with random roughness appears to be a practical candidate [122]. Wang and Kang [122] generated 3D randomly generated microporous media with random roughness on a microchannel. They set three parameters: number density, total volume fraction, and anisotropy of the roughness elements. Figure 32 shows the microchannel with different roughness. For details regarding roughness generation, refer to Wang and Kang's work [122]. The EOF rate in this work was defined as

(64) $$Q\;=\int u_{z}dA,$$

where $u_{z}$ represents EOF velocity in the applied external electric field direction and A is the area of the microchannel cross‐section. Figure 32A shows the normalized EOF rate versus the roughness number density of the microchannel. The roughness number density is defined as

(65) $$n_{R}=\frac{N_{wall}s_{d}}{A_{wall}},$$

where $N_{wall}$ and $A_{wall}$ are the total cell number and the area of the microchannel wall, respectively. Increasing the roughness number for a fixed microchannel walls’ area and the total cell numbers was carried out by increasing the roughness distribution probability. As we have shown in Fig. 32D–F, an increase in $s_{d}$ would make the roughness microstructure coarser with less connections. The impact of the roughness number density on the EOF rate is shown in Fig. 33A. It is interesting to note that the EOF rate increases linearly with the logarithm of the number density of roughness. These results indicate that a more connected or dense roughness of the microchannel will decrease EOF pumping effect. In Wang and Kang's work, the roughness volume fraction was defined as the ratio of the total volume of the roughness to the fluid volume for the microchannel with smooth walls. If we fix the number density of the roughness as $n_{R}\;$= 360/μm^2^ for the squares and $n_{R}\;$= 36/μm^2^ for circles in Fig. 33B, then the numerical modeling results revealed that the EOF rate generally decreases with increase in the volume fraction for both the number densities of the roughness scenario. To understand the impact of volume fraction on the EOF rate, we can imagine that increasing the volume fraction means that the total volume of the roughness increases for a fixed microchannel height.

These results are reasonable as by increasing the volume fraction of the roughness, the effective area for electrokinetic transport is decreased. Moreover, it has been shown that by increasing the number density of roughness, the EOF rate will increase. These results are consistent with the impact of the number density on the EOF rate (Fig. 33A).

**Figure 34:**
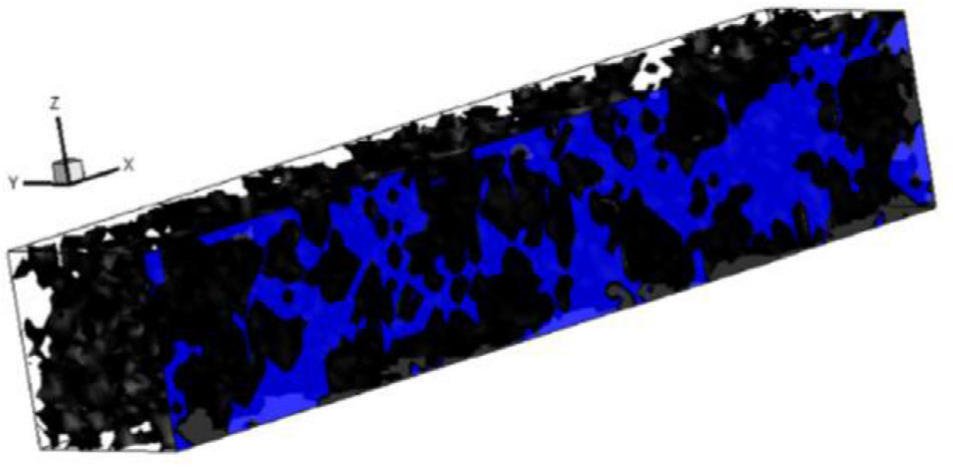
3D microporous medium that was generated by the random generation‐growth method. The black parts represent the solid and the blue parts are the voids filled by the solution. EOF will be in the *x* direction. This figure has been reprinted from [123].

The works discussed above (except Wang et al. [120] in which the solution pH determines the zeta potential and surface charge of solid‒liquid interface) investigated charged microporous media where the surface charge was prescribed and homogenous. This means that the surface charge was an input parameter and did not change with the solution properties. However, Zhang and Wang [123] investigated electroosmosis in an inhomogeneously charged microporous medium. An inhomogeneously charged porous medium refers to the fact that the surface charge on the solid–liquid interface must be obtained based on the local solution properties (i.e., pH, temperature, and bulk ion concentration). Therefore, one of the EDL theories that we introduced in Section 2.1 must be employed. Zhang and Wang [123] obtained the local surface charge by using a 1‐pK model, which is a simpler solution compared to complicated models such as the triple layer model [15, 47, 124] or the quad layer model [13, 17]. However, the details of the model that was used by Zhang and Wang is not the focus of this tutorial. By following the random generation‐growth method, they reconstructed a 3D microporous media as shown in Fig. 34. The external electric field was applied in the *x* direction and EOF was in this direction. The same governing equations as the previous works were solved, except the boundary condition for the Poisson's equation in which the zeta potential was $\psi_{S}=\;\zeta \left(x,y,z\right)$.

**Figure 35:**
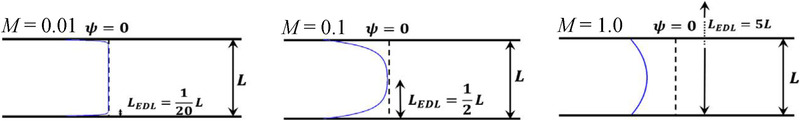
EOFs based on the EDL overlapping regimes. The blue lines represent the distribution of the electric potential owing to the charged solid–liquid interface. The figure has been reprinted from [123].

As we discussed above as well, one challenging fact about EOF through straight channels or the porous media is to figure out the overlapping of EDLs. Therefore, it is essential to realize when the overlapping happens. Therefore, Zhang and Wang [123] proposed a parameter inspired by the Knudsen (Kn) number. Generally, the Kn number is defined as the ratio of the mean free path of the molecules to the characteristic length of the space, which could be the channel height or the pore diameter. This number determines whether continuum mechanics or statistical mechanics must be utilized to model the fluid mechanics. Inspired by the Kn number, they proposed the M number, which is equivalent to the ratio of the EDL thickness to the characteristic length of the domain M=λ/L. Based on this number, EOF can be divided into four regimes as (Fig. 35)

(66) $$\begin{array}{c} •\;\mathrm{Thin}\;\mathrm{layer}\;\mathrm{regime}:M\leq 0.01,\\ •\;\mathrm{Non}-\mathrm{overlapped}\;\mathrm{layer}\;\mathrm{regime}:0.01<M\leq 0.1,\\ •\;\mathrm{Partially}\;\mathrm{overlapped}\;\mathrm{layer}\;\mathrm{regime}:0.1<M\leq 1,\\ •\;\mathrm{Fully}\;\mathrm{overlapped}\;\mathrm{layer}\;\mathrm{regime}:M>1. \end{array}$$

As Fig. 35 demonstrates, by increasing the EDL thickness, most of the channels’ bulk will be charged and influenced by the charge of the solid–liquid interface. It is worth pointing out that any further increase of the EDL thickness, which can be done by decreasing the characteristic length of the domain or decreasing the bulk ionic concentration, will lead to monotonic distribution of the electric potential across the channel or pore at M≫1. Therefore, we can assume that the electrical charge of the whole channel or pore is identical to the charge of the solid–liquid interface.

**Figure 36:**
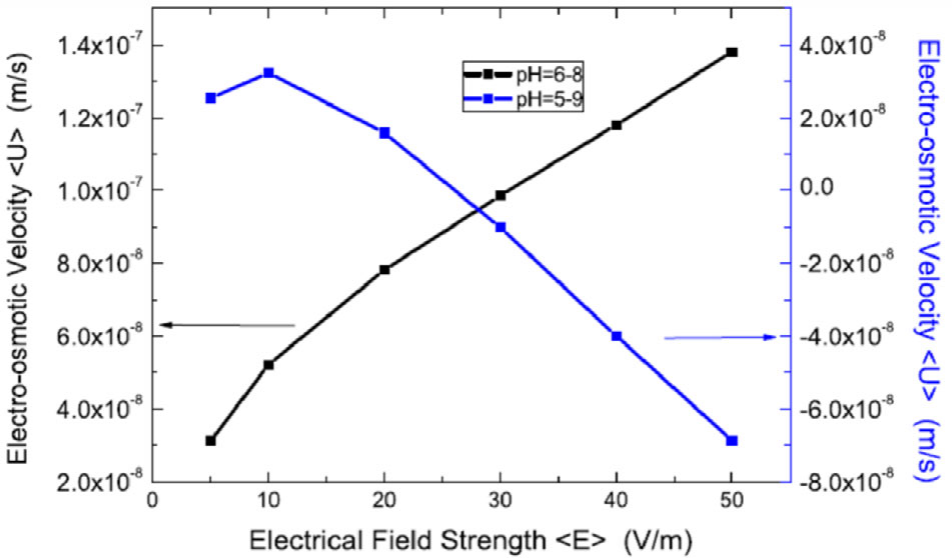
EOF velocity through the porous media with porosity 0.46. To induce surface charge inhomogeneity, two pH gradients were employed in which the black symbol line demonstrates the pH from inlet to outlet identical to 6 and 8 and the red symbol‐line demonstrates the pH from inlet to outlet equal to 5 and 9. These results have been reprinted from [123].

To investigate the effect of the applied external electric field and the inhomogeneity of the surface charge on EOF, the averaged EOF velocity for different applied electric field strength and two pH gradients were studied for a porous media with porosity ε=0.46 (Fig. 36). The modeling results show that for E<20 V/m, EOF shows a nonlinear behavior with respect to the applied electric field. This effect could be interpreted by considering the fact that the diffusivity of the hydrogen (H^+^) and the hydroxyl (OH^−^) ions are different. When we increase the applied external electric field, the movement of hydrogen ions toward the outlet will be greater, and the local distribution of the pH will be affected by the applied external electric field. While the local pH distribution close to the outlet decreases, as we know, the local absolute zeta potential will decrease. The interplay of the applied external electric field and lower influence from the solid–liquid interface at the outlet of the porous media will generate a nonlinear behavior of EOF velocity. However, by increasing the applied external electric field, the impact of the electromigration phenomenon will be dominant. As a result, EOF velocity can be determined by a linear relationship with E (see Fig. 36 for E>20 V/m).

## 4 EOF through nanoscale pores and channels

In the previous section, we discussed EOF and its application when the characteristic length of the domain is on the order of a few micrometers. This scale explained how the solution could be transferred through nonoverlapped EDL regimes. We introduced different practical applications, including micromixing, EOF through porous media with applications in energy (e.g., enhanced oil recovery) and environment (underground water remediation), as well as two methods to characterize the electric charge at the solid–liquid interface. The first one, which is generally employed for the sake of simplicity, prescribes the surface charge as a spatially nonvariable parameter while the second one obtains the local surface charge based on the local solution properties. Clearly, the second strategy is more realistic in considering the effects of local solution property on EOF in the presence of applied pH or concentration gradients. In this section, we introduce EOF through straight channels and porous media for characteristic lengths on the order of a few nanometers. At this scale, the transport medium guarantees overlapping of the EDLs, due to which interesting phenomena begin to emerge.

### 4.1 EOF through a straight nanochannel

As briefly alluded to earlier, we are interested in studying EOF through nanometer sized channels due to the strong overlapping of the EDLs. This results in a nonuniform velocity distribution along the nanochannel's height due to the strong interaction of the major solution with the applied external electric field [125]. There is a large body of literature that investigates the physics underlying the transport of ionic species through confined domains such as nanofluidic channels [125, 126, 127, 128, 129, 130, 131, 132, 133, 134]. However, we aim to focus on the fundamentals of electroosmosis and the impact of the solid–liquid surface charge on EOF.

**Figure 37:**
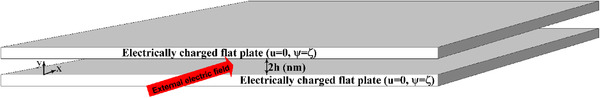
Schematic illustration of the nanochannel, considered to be the space between two parallel flat plates that are equally charged. The external electric field is applied to the *x* direction. The height of the nanoslit is 2h.

Historically, Burgreen and Nakache [2], for the first time, developed the theory of electrokinetic flow in ultrafine capillary slits. They extended the general theory of the electrokinetic flow, which was earlier limited to the nonoverlapped regime of EDLs. Later, Levine et al. [135] extended Burgreen and Nakache's work in which the charge regulation phenomenon due to the overlapping of EDLs was taken into account. Generally, charge regulation is attributed to the surface charge variation owing to reduction in distance between two charged plates [136]. This phenomenon is usually observed in overlapped EDL regimes where the conduction current exceeds the conduction in the bulk solution [137]. Furthermore, Levine et al. [135] considered the convection current generated due to the applied external electric field. Following Levine et al. method, we are going to introduce the semianalytical solution for EOF through a confined channel, which is considered to be the space between two parallel symmetrically charged infinitely big flat plates (Fig. 37).

EOF will be investigated as a function of the applied external electric field in the *x* direction, which is parallel to the flat plate surface. It is assumed that the solution is under no‐slip boundary conditions on the surface of the plates (y=0). Considering steady‐state conditions, Eq. (53) reduces to

(67) $$\mu \frac{d^{2}u\left(y\right)}{dy^{2}}+\rho_{e}\left(y\right)E-\;\frac{dp}{dx}=\;0,$$

where due to the symmetry of the system in the *y* direction, Eq. (67) could be solved for the half‐domain as 0≤y≤h. The boundary conditions for the NS equation (Eq. (67)) are

(68) $$u\left(y\;=\;0\right)=\;0,\;\;\frac{du\left(y\right)}{dy}{|}_{y\;=\;h}=\;0.$$

Here similar to what we did for the microchannel, the impact of external electric field and the EDL electric field is decoupled, which gives rise to

(69) $$\Psi \;=\;\psi -EL_{p},$$

where $L_{p}$ denotes the length of the plate in the *x* direction. It must be noted that this decoupling of the electric potential is only valid when the applied external electric field is small [135] or when ignoring the entrance effects. This assumption will let us consider the total electric potential as a superposition of the applied external electric field and the electric potential owing to the electrically charged solid–liquid interface. Consequently, we can easily solve Poisson's equation for ψ as well. The boundary conditions for Poisson's equation are proposed as

(70) $$\begin{array}{cc} & \psi_{y\;=\;0}=\;\zeta ,\\ & {\left.\frac{d\psi_{y=0}}{\mathrm{dy}}\right|}_{y\;=\;h}=0. \end{array}$$

If we substitute Eq. (1) into Eq. (67), then we have

(71) $$\mu \frac{d^{2}u\left(y\right)}{dy^{2}}-E\varepsilon \varepsilon_{0}\frac{d^{2}\psi_{y\;=\;0}}{dy^{2}}-\;\frac{dp}{dx}=\;0.$$

If we introduce the velocity and the electric potential boundary conditions into Eq. (71), then we have an analytical solution for EOF velocity as

(72) $$u\left(y\right)=\;-\frac{P}{2\mu }\left(y^{2}-2hy\right)+\frac{E\varepsilon_{r}\varepsilon_{0}}{\mu }\left(\psi \left(y\right)-\zeta \right),$$

where $P\;=\;-\frac{dp}{dx}$ denotes the applied pressure gradient. Equation (72) indicates that the total electrolyte velocity is a result of both pressure‐driven and electroosmotic fluid flow. While we are interested in EOF velocity, we can set P=0, which Eq. (72) gives rise to

(73) $$u_{EOF}\left(y\right)=\frac{E\varepsilon_{r}\varepsilon_{0}}{\mu }\;\left(\psi \left(y\right)-\zeta \right).$$

It is interesting to note that Eq. (73) reduces to the HS velocity if we assume that 2h≫λ. Based on this assumption, we can assume that a major part of the channel will not be electrically charged and, as a result, we have ψ(y)≈0. Hence, Eq. (73) reduces into HS velocity $U_{HS}=\;-\frac{E\varepsilon_{r}\varepsilon_{0}}{\mu }\zeta$. By introducing $U_{HS}$ into Eq. (73), we have

(74) $$u_{EOF}\left(y\right)=U_{HS}\;\left(1-\frac{\psi \left(y\right)}{\zeta }\right).$$

If we are interested in the mean EOF along the nanoslit cross‐sections, we can simply integrate both sides of Eq. (74), which gives rise to [2, 135]

(75) $${\bar{u}}_{EOF}=U_{HS}\;\left(1-G\right),$$

where G is defined as

(76) $$G\;=\;G\;\left(\zeta ,\kappa h\right)=\frac{1}{h\zeta }\;\int_{0}^{h}\psi \left(y\right)dy\;=\frac{\bar{\psi }}{\zeta },$$

where the function G represents the ratio of the mean electric potential to the electric potential (zeta potential) of the solid–liquid interface.

Thus far, we introduced an analytical solution for EOF velocity through a confined nanoslit. However, the only remaining challenge is to obtain the distribution of the electric potential along the cross‐section of the nanoslit. The Poisson–Boltzmann equation could be utilized for this specific problem, where ψ could be obtained as

(77) $$\frac{d^{2}\psi^{*}}{d{y^{*}}^{2}}=\frac{1}{2}\;\left(\exp\left(\psi^{*}\right)-\exp\left(-\psi^{*}\right)\right),$$

where $\psi^{*}$ and $y^{*}$ are the dimensionless formats of the electric potential, defined as $\psi^{*}=\;\psi /V_{T}$ and the unit of length as $y^{*}=\;y/\lambda$, respectively. Equation (77) could be rewritten in the form of

(78) $$\frac{d^{2}\psi^{*}}{d{y^{*}}^{2}}=\sinh\left(\psi^{*}\right).$$

Levine and Suddaby [138] demonstrated that there is an analytical solution to the rapidly converging series for the electric potential as

(79) $$\psi^{*}\;\left(y^{*}\right)=\;8\sum_{r\;=\;0}^{\infty }\frac{q^{{}^{\prime }\left(2r+1\right)}}{1-q^{{}^{\prime }\left(4r+2\right)}}\frac{\cosh\left[\left(2r+1\right)\left(1-y^{*}\right)v\right]}{2r+1},$$

where $k^{\prime }={\left(1-k^{2}\right)}^{0.5}\;$, K=K(k), $v\;=\frac{\pi \kappa h}{2k^{0.5}K^{\prime }}\;$, $q^{\prime }=\exp\left(-\frac{\pi K}{K^{\prime }}\right)\;$, and $K^{\prime }=\;K\left(k^{\prime }\right)$ where k is defined as $k\;=\exp(-\psi^{*}\left(h\right))\;$ and $K\;\left(k\right)=\;F\left(\frac{\pi }{2},k\right)$. F is the incomplete elliptic integral of the first kind and defined as [135]

(80) $$F\;\left(\phi ,k\right)=\int_{0}^{\phi }\frac{d\theta }{{\left(1-k^{2}\sin^{2}\theta \right)}^{0.5}}.$$

If we introduce Eq. (79) into Eq. (76), then we have a relation for G as

(81) $$G\;=\frac{8}{\psi^{*}\left(0\right)}\;\sum_{r\;=\;0}^{\infty }\frac{q^{{}^{\prime }\left(2r+1\right)}}{1-q^{{}^{\prime }\left(4r+2\right)}}\frac{1}{2r+1}\frac{\sinh\left[\left(2r+1\right)v\right]}{\left(2r+1\right)v},$$

where

(82) $$\psi^{*}\;\left(0\right)=\;8\sum_{r\;=\;0}^{\infty }\frac{q^{{}^{\prime }\left(2r+1\right)}}{1-q^{{}^{\prime }\left(4r+2\right)}}\frac{\cosh\left[\left(2r+1\right)v\right]}{2r+1}.$$

Thus far, we introduced an analytical solution for EOF velocity and the electric potential when the electrolyte is moving through a nanoslit under application of an external electric field. In another attempt to characterize electroosmosis through nanochannels, Pennathur and Santiago [125] proposed an analytical solution for EOF and experimentally validated their theoretical study [126]. Regarding the theoretical work, the NS equations that demonstrate the balance of the viscous flow and the applied Lorentz electric force on the solution were considered to be

(83) $$\begin{array}{c} \nabla \cdot u=0,\\ \mu \nabla^{2}u=-\rho_{e}\nabla \Phi , \end{array}$$

where μ is the dynamic viscosity and Φ denotes the total electric potential, which could be decoupled (following Probstein [139]) to the external electrical potential due to the applied electric field and the internal electric field because of the EDL effect as Φ=ϕ+ψ. Integration of Eq. (83) gives rise to Eq. (74). By double integration of Eq. (74) across the cross‐section of the nanochannel, Pennathur and Santiago [125] obtained the area‐averaged velocity by numerical simulation. However, an analytical solution for low zeta potentials is available based on the Debye–Hückel theory [139]:

(84) $$u_{EOF}\left(y\right)=U_{HS}\;\left(1-\frac{A_{1}\exp\left(\frac{y}{\lambda }\right)+A_{2}\exp\left(-\frac{y}{\lambda }\right)}{\zeta }\right),$$

where

(85) $$\begin{array}{c} A_{1}=\zeta \left(\frac{1-\exp\left(\frac{2h}{\lambda }\right)}{\exp\left(\frac{h}{\lambda }\right)-\exp\left(-\frac{3h}{\lambda }\right)}\right),\\ A_{2}=\zeta \exp\left(-\frac{h}{\lambda }\right)-A_{1}\exp\left(-\frac{2h}{\lambda }\right). \end{array}$$

Here it is worth reminding that EOF that is proposed by Eq. (84) is only valid for very small zeta potentials when $\zeta \ll V_{T}$.

**Figure 38:**
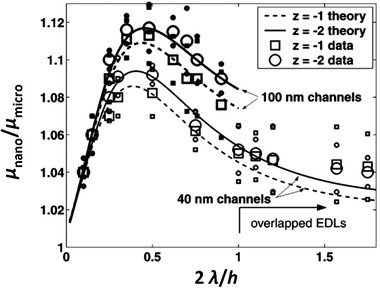
Normalized analyte ion mobility as a function 2λ/h in nanochannel with the mobility in a microchannel. The normalized mobility is compared with the theoretical model. The large open symbols are the measured data and the closed symbols are the error bars. The overlapping of the EDL is for $\frac{2\lambda }{h}>1$. The measurements were performed for two nanochannels with 40 and 100 nm height. Reprinted with permission from [126]. Copyright (2005) American Chemical Society.

One way to analyze EOF through the nanofluidic channels is to consider the electrolyte solution with some analyte ion. For instance, let us assume an electrolyte solution with fully ionized background electrolyte with ionic species *A* and *B*. The general NP equation with electromigration‐diffusion‐advection contributions can predict the distribution of all the ionic species. As a low concentration of the analyte ion *S* does not affect the distribution of background ionic species, we can separate the governing equation for background ionic species *A* and *B* from the analyte ion *S*. With this aim, Pennathur and Santiago [125] developed a relation to explain the analyte ion velocity as

(86) $$\begin{array}{ccc} \left\langle u_{S}\right\rangle & = & \left\langle u_{EOF}\right\rangle +\left\langle u_{EP}\right\rangle \\ & = & -U_{HS}\langle \exp\left(-z_{S}e\left(\frac{\psi \left(y\right)-\psi_{c}}{k_{B}T}\right)\right)\left(1-\frac{\psi \left(y\right)}{\zeta }\right)\rangle \\ & & +\;\nu_{S}z_{S}FE, \end{array}$$

where $\psi_{c}$ represents the centerline of the nanochannel electric potential. $z_{S}$, F, and $\nu_{S}$ represent the ionic valence of the sample analyte, Faraday's constant, and the ion mobility, respectively. Equation (86) denotes the average velocity of the sample analytes as a result of EOF flow due to the background solution movement together with the electrophoresis movement of the sample analytes due to the applied external electric field. Figure 38 shows the normalized mobility of the ionic species in a nanochannel with respect to the mobility in microchannel as a function of 2λ/h. It is interesting to note that mobility in both nanochannels with 40 nm and 100 nm height, increases with increase in the thickness of EDL. However, the increase in ionic mobility has a peak at 2λ/h≈0.5 and any further increase in EDL thickness (i.e., stronger overlapping of EDLs) decreases mobility. One reason behind this is that upon an increase in overlapping of EDLs, the first term on the right‐hand side of Eq. (86) tends to zero because the electric potential in the bulk solution tends closer to the zeta potential $(1-\frac{\psi (y)}{\zeta })\to 0$. Consequently, the electrophoretic phenomenon plays a key role in driving the species from the inlet to the outlet of the nanochannel.

The electroosmosis inside the nanochannel could be employed to separate the species with a different electrical charge. As Eq. (86) indicates, the positive sample analytes experience lower velocity in comparison with the negative analytes. This simple fact was used by Garcia et al. [140] to demonstrate molecular separation in nanoscale fluidic channels. They analyzed the electrokinetic transport of the negatively charged species by introducing a negatively charged dye, Alexa 488, and EOF with a neutral dye, rhodamine B. Their measurements revealed that for a negatively charged nanochannel, the electroosmotic velocity toward the cathode (negative electrode) of the negatively charged dye is higher than the neutral dye. This behavior of the negatively charged species inside a nanochannel is anomalous. It is the opposite of what was observed with microchannels, where the neutral dye was transported faster than the negatively charged dye. The main reason for this phenomenon is that for the microchannel, the electrophoretic drag force will decrease the speed of the negatively charged species while the neutral species move faster. This interesting experiment demonstrated the impact of the transport phenomena in nanochannels with overlapped EDLs and microchannels with nonoverlapped EDL regimes. To explain their experimental measurements, the authors followed the same theory as we discussed above for EOF through nanochannels.

The same relation as Eq. (74) for EOF velocity was proposed while the local electric potential was obtained analytically as [39]

(87) $$\begin{array}{ccc} \psi^{*}\;\left(y\right) & = & \frac{4}{z}\;\left(\tanh^{-1}\left(\tanh\left(\frac{\zeta^{*}}{4}\right)\exp\left(-\kappa y\right)\right)\right)\\ & & +\frac{4}{z}\left(\tanh^{-1}\left(\tanh\left(\frac{\zeta^{*}}{4}\right)\exp\left(-\kappa \left(h-y\right)\right)\right)\right), \end{array}$$

where $\psi^{*}$ is an approximate solution for the electric potential under three assumptions: (I) 2D flow in (II) channels with parallel walls and (III) weak EDL interaction. By introducing the electric potential (Eq. (87)) into Eq. (74), Garcia et al. [140] obtained the concentration‐weighted velocity of a dye as a function of EOF and electrophoretic velocities:

(88) $$u_{tot}=\frac{C\left(y\right)u_{EOF}\left(y\right)}{\bar{C}}\;-u_{ep},$$

where $\bar{C}=\frac{1}{h}\;\int_{0}^{h}C\left(y\right)dy$ and $C\;\left(y\right)=n_{b}\;\exp\left(-z\psi^{*}\left(y\right)\right)$.

**Figure 39:**
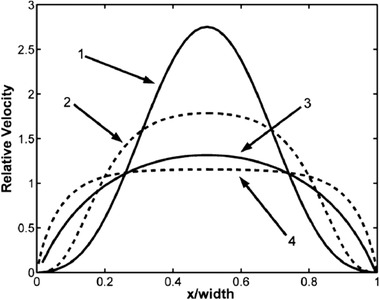
Dimensionless velocity because of the electroosmosis for the negatively charged dye in (1) 60 nm and (2) 200 nm height nanochannels. Curves (3) and (4) represent the 60 and 200 nm nanochannels for the neutral dye, respectively. The figure has been reprinted from [140].

As Fig. 39 demonstrates, on one hand, for the neutral species, there is no peak velocity in the middle of the nanochannel. This phenomenon is expected because the neutral species do not feel any electrostatic force from the charged solid–liquid interface. Consequently, there would be a uniform distribution of the neutral dye at the middle of the nanochannel and uniform concentration‐weighted velocity at the major part of the nanochannel (Fig. 39, curves (3) and (4)). In contrast, the negatively charged dye will mostly repel the middle of the nanochannel owing to the negative surface charge on the solid–liquid interface, which makes the $\bar{C}/C\left(y\right)$ smaller and consequently increases concentration‐weighted velocity at the centerline of the nanochannel (Fig. 39, curves (1) and (2)). Furthermore, Fig. 39 demonstrates the impact of the nanochannel height on the concentration‐weighted velocity. It shows that the maximum electrokinetic velocity differences will be in the approximately 60 nm height or when κh∼4. For heights below this number, the interaction of the EDLs will increase and, as a result, an electric potential along the cross‐section of the nanochannel will be developed to the zeta potential. This will lead to the development of EOF velocity profiles.

**Figure 40:**
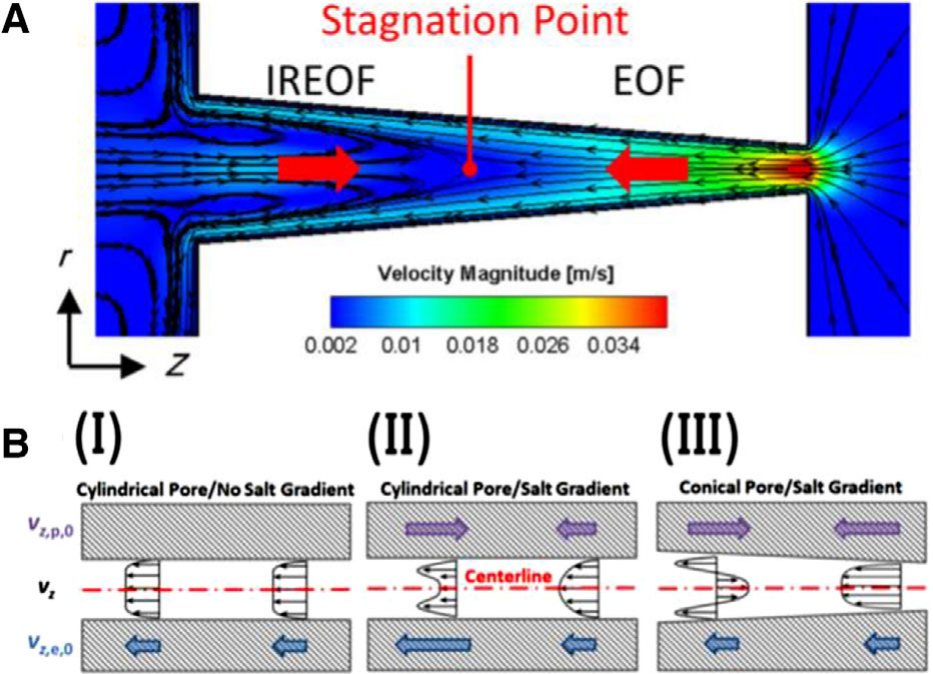
(A) Flow field in a negatively charged conical nanopore located between two large reservoirs carrying different salt concentrations when an axial electric field is imposed. (B) Schematics of EOF in (I) a cylindrical pore without a salt gradient, (II) a cylindrical pore with an axial salt concentration gradient, and (III) a conical pore with an axial salt concentration gradient. Reprinted with permission from [149]. Copyright (2018) American Chemical Society.

**Figure 41:**
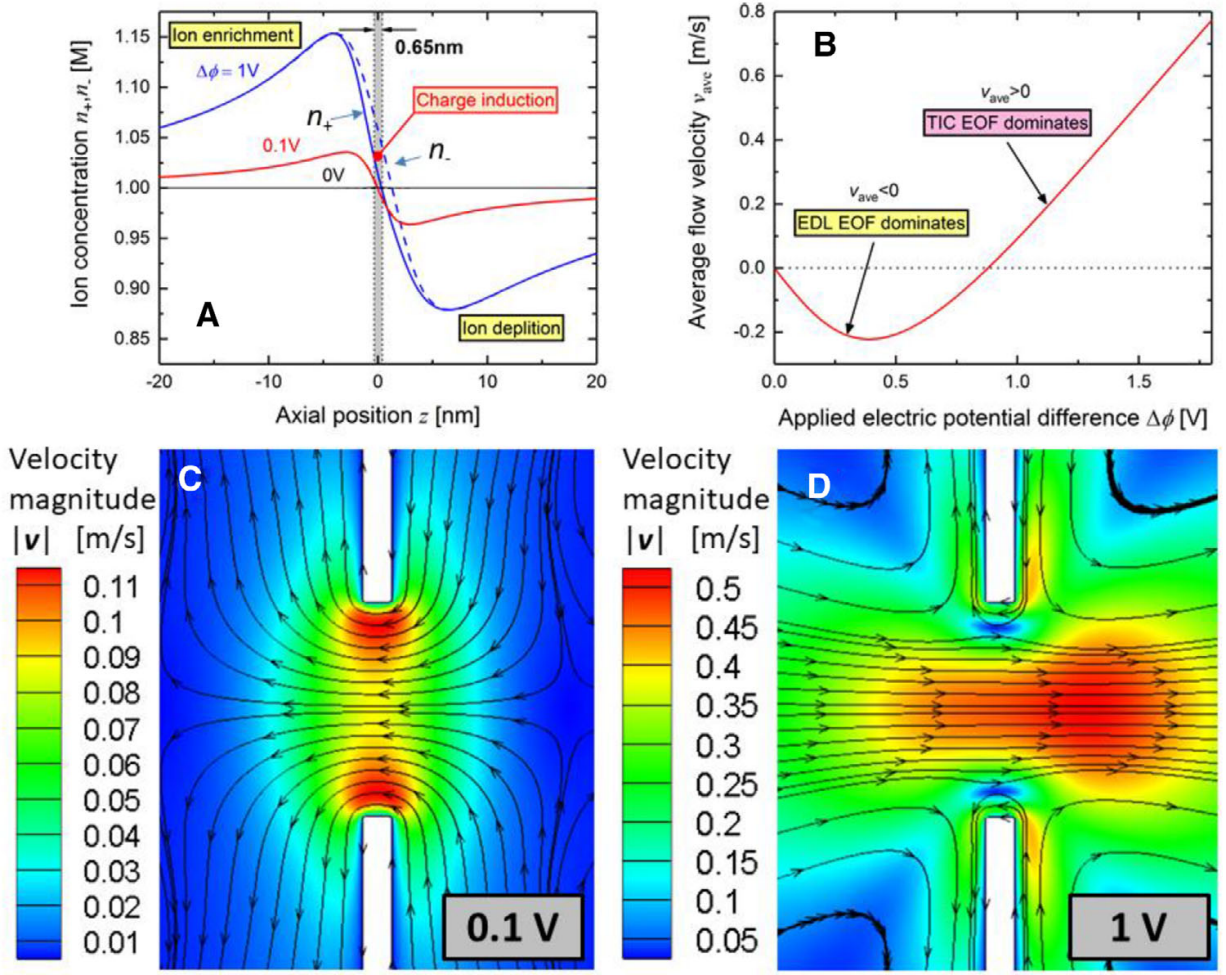
(A) Variation in ion concentration$\;n_{+}$, $n_{-}$ (solid and dashed curves indicate $n_{+}$ and $n_{-}$, respectively) along the monolayer molybdenum disulfide nanopore axis at different applied electric potential differenceΔϕ. (B) Variation in average flow velocity $v_{\mathrm{ave}}$ (negative and positive values indicate the solution directions toward the cathode and anode, respectively) as a function ofΔϕ. Contours of the flow velocity magnitude |ν| and streamlines in the monolayer molybdenum disulfide nanopore at the bulk concentration $n_{0}$ = 1 M, and (C) Δϕ = 0.1 V, (D) Δϕ = 1 V. The gray shadow area in (A) indicates the nanopore region and “EDL EOF” and “TIC EOF” in (B) denote the “electric double layer electroosmotic flow” and “transport‐induced‐charge electroosmotic flow,” respectively. Reprinted with permission from [149]. Copyright (2018) American Chemical Society.

The employment of a nonuniform EOF can enhance the efficiency of protein separation in nanochannels as well as the efficiency of molecule detection in nanopores. Considering a nonuniform zeta potential distribution along the surface of cylindrical capillaries by partially coating with a polymer layer, Herr et al. investigated the induced pressure gradient and nonuniform EOF profiles along the axial direction [141]. Similarly, distribution of surface charge and EDL thickness can be generated by introducing salt concentration or pH gradients in silica channels [142, 143, 144, 145], enabling high‐performance label‐free separation of proteins in nanochannels [146, 147]. In terms of nanopore sensing, it has been experimentally demonstrated that both capture rate and translocation time of DNA molecules in a nanopore can be simultaneously enhanced by adding a salt concentration gradient [148]. Hsu and Daiguji theoretically investigated electroosmotic behavior in a cylindrical nanopore channel when a salt concentration gradient exists in the axial direction [149]. Due to the variation in EDL thickness, a nonuniform EOF was induced along the surface, which yielded an induced pressure gradient near the nanopore centerline to satisfy mass conservation, as shown in Fig. 40. As a result, EOF became weaker near the molecule entrance (downstream), facilitating the capture, while the amplified EOF near the *trans* reservoir in the nanopore (upstream) extended the translocation time. Moreover, it was found that a conical geometry can enlarge this effect, giving rise to an induced reverse EOF (IREOF). This unique characteristic enables flexible control of flow behavior in a nanoconfined space for molecule manipulation in versatile bionanosensing systems by altering the bulk solution conditions. Not only can the concentration difference be directly generated by manipulating the bulk conditions, but a local concentration can be induced at the junctions between the nanochannels/nanopores and the microchannels (or reservoirs) when an electric field is applied over ion‐selective channels, due to ion concentration polarization [150]. As seen in Fig. 41A, a clear concentration difference occurs that increases with the magnitude of the applied electric field. The large electric field and concentration gradient across an ultrathin nanopore yield significant ion separation, known as transport‐induced‐charge (TIC) [151]. Note that both induced charge and surface charge are negative and, hence, a reversal of EOF appears when increasing the applied electric field, as shown in Fig. 41B. At low applied electric potentials, the EOF behavior is dominated by the charge at the interface (see Fig. 41C) whereas EOF direction along the axis is governed by TIC when the applied electric potential is high (see Fig. 41D).

This nonlinear electroosmotic behavior has an enormous influence on DNA molecule translocation behavior through 2D nanopores, where a threshold voltage is needed for a translocation event to occur to overcome the opposite EOF [152]. An ion concentration difference between the nanopore inlet and outlet could be induced when an electric field exists within a nanochannel. The phenomenon is known as ion concentration polarization [153]. When a current is established in a negatively charged ion‐selective nanopore, cations at the anode end are transported through the nanopore to the cathode end. Anions at the cathode end are repelled from the electrode and accumulate at the nanopore junction. Conversely, ion concentration at the anode end near the nanopore junction decreases. As a result, a solute concentration difference between the nanochannel junctions is established.

### 4.2 EOF through nanoporous media

In the previous section, we introduced the theory of EOF through nanofluidic channels and discussed some relevant applications. We showed that the height of the channel plays a key role in multiphysics transport phenomena and that EOF velocity depends on the ratio of the nanochannel height to the EDL thickness. In this section, we discuss transport phenomena through unstructured media, such as nanoporous membranes and rocks, which has practical applications in water treatment [154, 155, 156, 157], underground remediation of toxic ionic species [26, 158], and biological applications [159].

**Figure 42:**
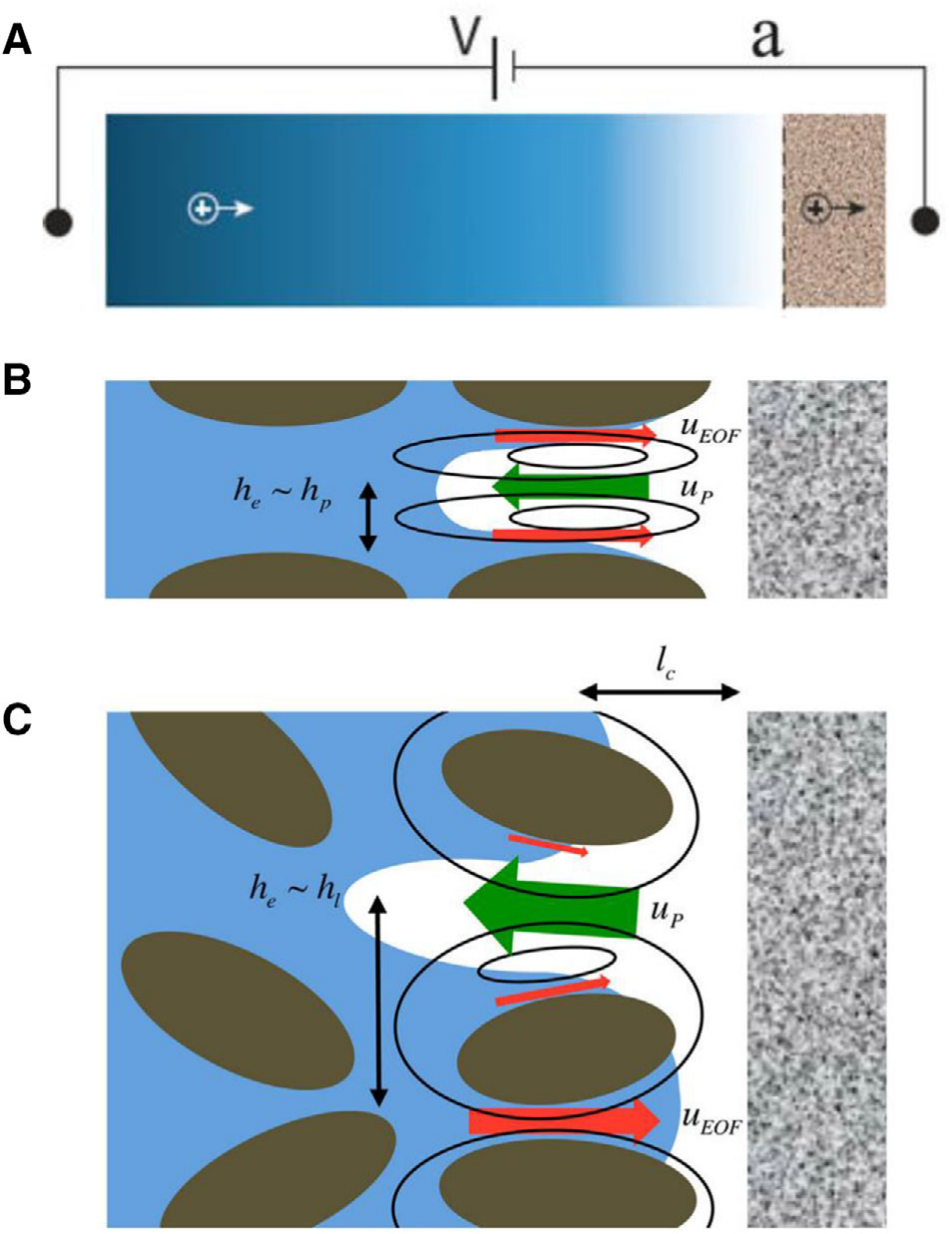
(A) Schematic illustration of the experimental setup in which the ionic current is driven from an anode in a reservoir to a perm‐selective membrane. (B) For the ordered porous media in the vicinity of the perm‐selective membrane, the strong EOF (red arrows) and back pressure‐driven (green arrows) electrolyte solution will initiate a salt (blue area) depletion region (white area) and the vortices are restricted to the space between grains. (C) For the porous media with a random distribution of the grains, the vortices are generated around the grains. Reprinted with permission from [160]. Copyright (2013) American Chemical Society.

The transport of ionic species through nanoporous media is of great interest theoretically and experimentally. For instance, Deng et al. [160] investigated the flow through a silica glass frit, which is a negatively charged porous medium. In their experimental study, the ionic current was driven from an anode in a reservoir to a perm‐selective (or cation‐selective) membrane, namely Nafion (Fig. 42A). The experimental setup was a sandwich of a reservoir, a 1 mm silica glass frit with an average pore size of 500 (nm), and a cation‐selective membrane. They stated that the transport of ionic species through an electrically charged nanoporous media is under the impact of the surface charge and EOF (Fig. 42B and C). Considering surface conductance (SC), Deng et al. [160] showed that the transport of the ionic species is mainly carried out by the surface charge for ultra‐confined porous membranes or low salinity electrolytes. This means that for the strong EDL overlapping regime, the surface charge determines the transport of ionic species through the porous media because the pores are electrically charged and makes it a counter‐ion‐selective medium.

**Figure 43:**
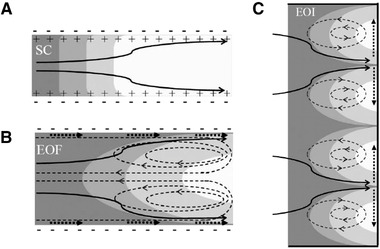
Three regimes of ionic species transport through a microchannel, which is dead ended via a perm‐selective nanoporous membrane. For further details see [161].

However, for the weak EDL interactions that could occur at larger pore sizes or more concentrated solutions, the transport of the ionic species is mainly carried out via EOF while the conductivity of the bulk solution is considerably higher than the EDL conductivity. This principle was first predicted theoretically by Dydek et al. [161]. They showed that the transport of the ionic species is mainly a function of the connected microchannel to the impermeable membrane. They found that the height (depth) of the connecting microchannel to the impermeable porous membrane specifies the ionic transport regime, which could be the SC (Fig. 43A), EOF (Fig. 43B), or electroosmosis instability (EOI) (Fig. 43C). For further useful information, refer to Dydek et al. [161]. We mentioned this phenomenon to emphasize the interesting phenomena observed at the interface of the nanoporous membrane and a microchannel.

**Figure 44:**
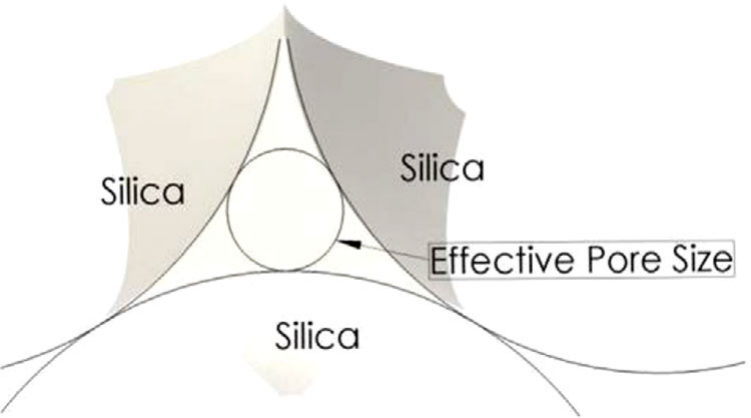
Schematic illustration of the effective pore size of the pack of solid silica nanospheres. The figure has been reprinted from [162].

EOF through the nanoporous media could be characterized by introducing a fluorescent buffer solution to examine EOF velocity through nanoporous media with pores on the order of the EDL. With this aim, Bell et al. [162] carried out experimental measurements on EOF velocity for a nanoporous membrane made as a pack of solid silica nanosphere with effective pore sizes from 104 nm down to 8 nm (Fig. 44).

**Figure 45:**
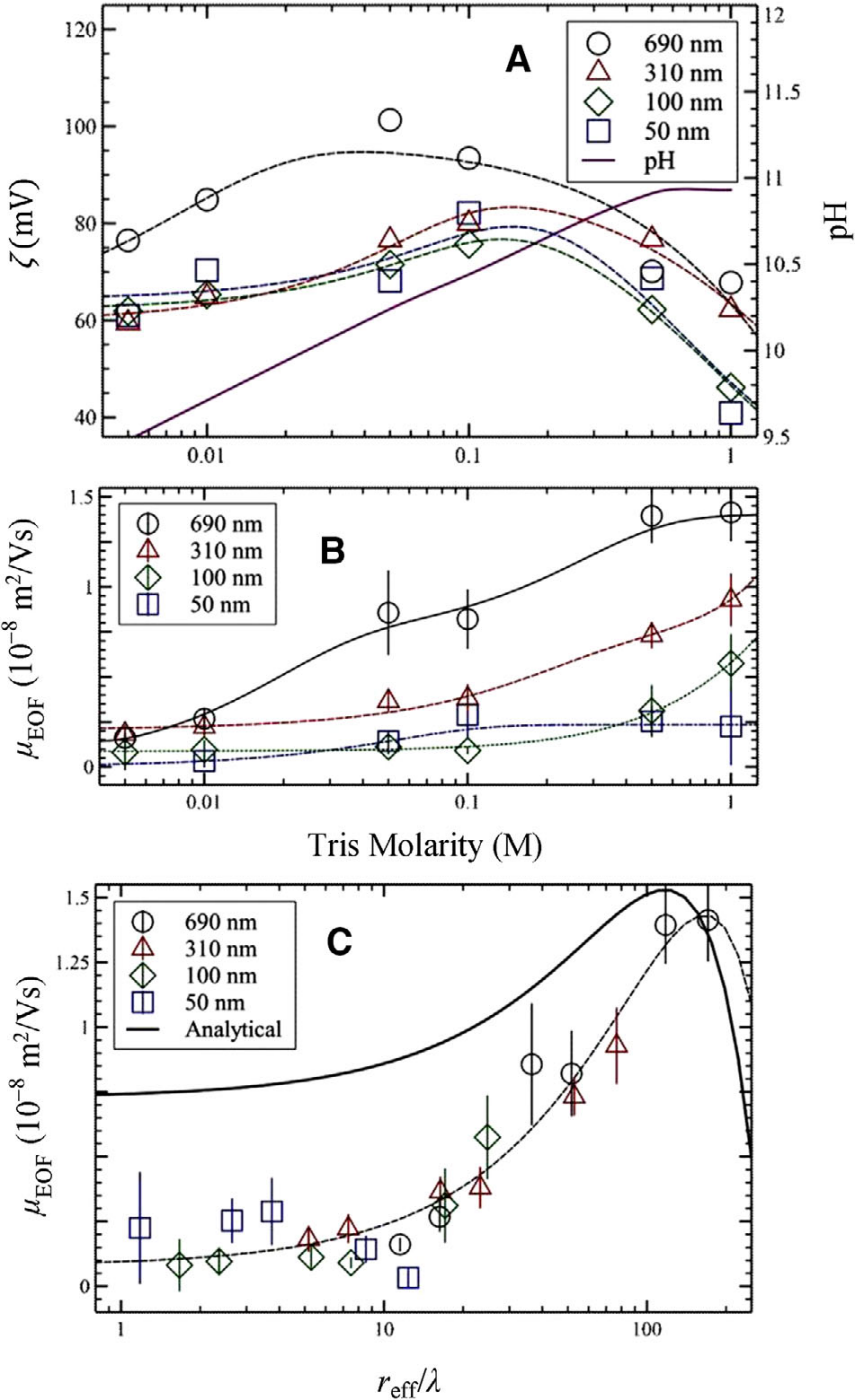
(A) Measured zeta potential as function of Tris molarity for the packed silica nanospheres. EOF mobility as a function of (B) Tris molarity and (C) the ratio of the effective pore size to the EDL thickness. The experimental measurements carried out for a different pack of solid silica nanospheres with distinct average pore sizes. The results have been reprinted from [162].

They utilized theoretical EOF mobility, which was defined by Ref. [163] as

(89) $$\mu_{EOF}=\left(1+\frac{\phi }{2}\right)\;\left(\frac{2\varepsilon \zeta }{3\mu }+\frac{\varepsilon \psi_{s}\chi }{3\mu }\right),$$

where $\mu_{EOF}$, ϕ, ε, μ, $\psi_{s}$, and χ denote EOF mobility, the volume fraction, electrical permittivity of the solution, dynamic viscosity, electric potential on the solid surface (which is considered to be the zeta potential), and the correction factor (which is a function of $\frac{r_{eff}}{\lambda }$), respectively. According to Levin et al. [5], for thin EDLs or larger effective pore sizes, χ=1; this means that EOF mobility will be solely a function of zeta potential. However, for thicker EDLs or smaller effective pore sizes, it can be determined in a way to consider the influence of the EDL overlapping on EOF mobility. Here, we should point out that EOF mobility is defined as the ratio of the average EOF velocity through the nanoporous media to the applied external electric field $\mu_{EOF}=\frac{u_{EOF}}{E}\;$. Levin et al.'s experimental measurements revealed that EOF mobility is not only increased by increasing the Tris concentration but also by the effective radius (Fig. 45B). Moreover, they showed that EOF mobility for all nanoporous media with distinct effective pore sizes will be reduced to a single curve if we plot EOF mobility as a function of $r_{eff}/\lambda$. This behavior of silica nanoporous media means that EOF mobility not only depends on the surface potential but also the overlapping of the EDLs [162]. EOF mobility, which was predicted by Eq. (89), is supported by experimental data Fig. 45C) where zeta potential of the packed silica nanospheres was measured for different packs (Fig. 45A).

**Figure 46:**
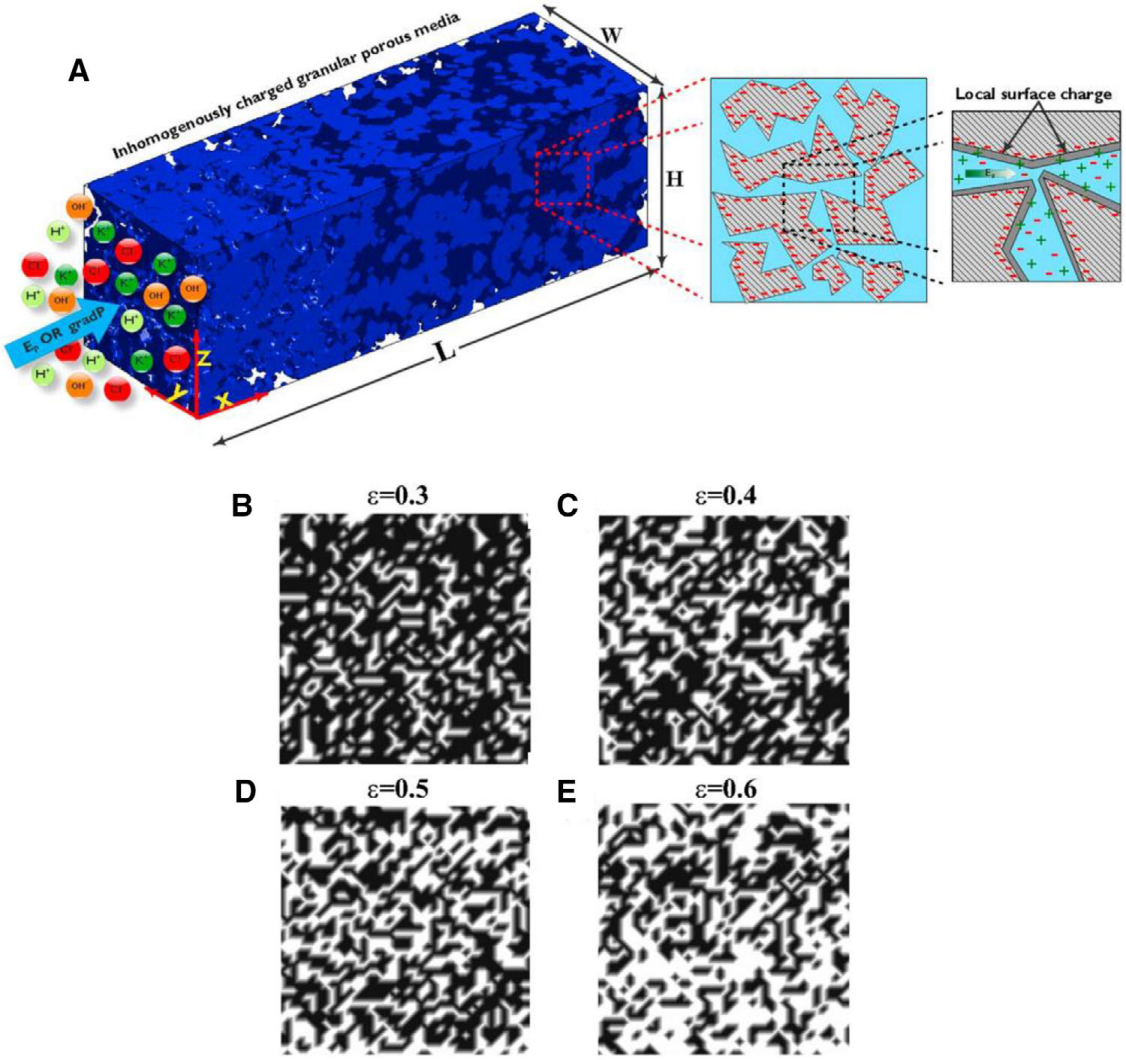
(A) Schematic illustration of the 3D randomly generated porous media where four different ionic species are introduced at the inlet of the porous media. The cross‐sections of the porous media are shown with porosities (B) ε= 0.3, (C) ε= 0.4, (D) ε= 0.5, and (E) ε= 0.6. The figures have been reprinted from [96].

Similar to what we discussed for ionic transport through microporous media (Section 3.2), transport through nanoporous media can be investigated by considering the local solution properties and, consequently, the local surface charge impact on EOF. As we discussed above, the experimental study has shown that transport in porous media with an overlapped regime is considerably different from that in nonoverlapped EDL regime that is typically found in microporous media. With this aim, Alizadeh et al. [96] conducted a theoretical study to investigate effect of local solution properties on the macroscopic transport phenomena in tight porous media with an average pore size on the order of a few nanometers. Three‐dimensional unstructured porous media (Fig. 46A) were generated via random generation‐growth method that we discussed in Section 3.2 with four porosities ε= 0.3, 0.4, 0.5, and 0.6 (Fig. 46B–E). Multicomponent ionic species were introduced at the inlet of the nanoporous media, which could be transferred from the inlet to outlet by applying an external electric field. To obtain EOF, it is essential to solving the Poisson, NP, and NS equations together. The impact of the local surface charge was incorporated into Poisson's equation as a boundary condition by employing the ETL model (Section 2.1).

**Figure 47:**
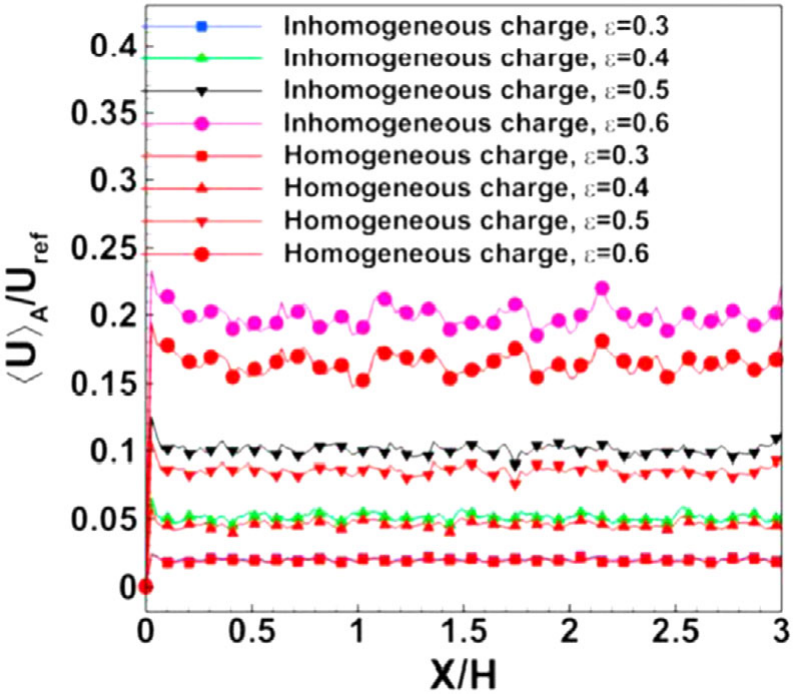
Normalized cross‐sectional averaged velocity along the length of the porous media. Two scenarios were compared: homogenous and inhomogeneous external charge distribution. The figure has been reprinted from [96].

Their modeling results showed a non‐negligible impact of the local surface charge on different aspects of transport phenomena through the nanoporous media. As the focus of this tutorial is on EOF, we only present EOF velocity along with the porous media (Fig. 47). As shown in Fig. 47, the inhomogeneous charge distribution and the porosity of the porous media significantly affect EOF velocity. It was shown that by increasing the porosity of the nanoporous media, EOF velocity increases for both homogeneous and inhomogeneous surface charge scenarios. However, EOF velocity is higher for the inhomogeneous scenario compared to the homogeneous scenario.

**Figure 48:**
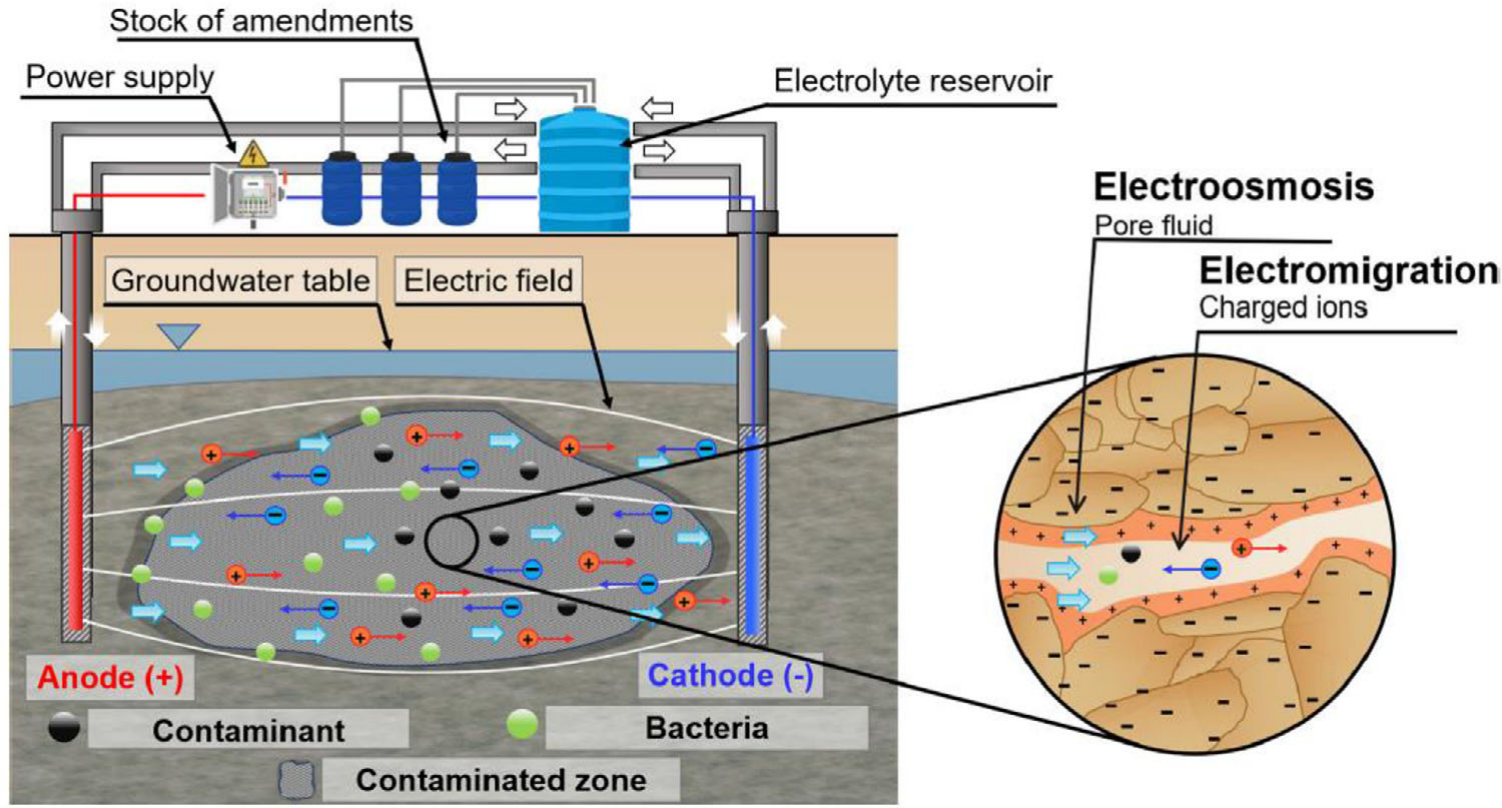
Schematic illustration of *in situ* EKR of groundwater from biological contaminants. The two transport mechanisms shown here are (I) electromigration and (II) electroosmosis. The figure has been reprinted from [165].

As we mentioned at the beginning of this section, one of the interesting applications of EOF is in driving underground water from one site to another desired site. Underground solute transport via EOF is one of the great advantages of this transport phenomenon. The principle of underground EOF is simple and straightforward. By applying an electric field, which only needs injected electrodes into the desired sites, we can collect the hazardous contaminants (i.e., arsenic) to a specified place and then remove them completely. This method, called EKR, has drawn considerable attention in recent years because of its ability in removing both organic and inorganic contaminants in low‐permeability domains such as underground soil [164]. There is a large body of literature that focuses on EKR. Its mechanism is the same as what we discussed for channels and porous media, which are based on (I) electromigration (displacement of charged species) and (II) electroosmosis (movement of a fluid flow owing to the nonzero net movement of the ionic species) (Fig. 48). A mechanistic understanding of the multidimensional transport of organic and inorganic contaminants coupled with the reactions in porous media has drawn attention recently [165, 166, 167]. In this regard, Sprocati et al. [166] modeled the electrokinetic transport with biogeochemical reactions in porous media for continuum scale by combining COMSOL Multiphysics and PhreeqcRM. The former was used for solving the Poisson–Nernst–Planck equations and the latter [168] for solving the geochemical reactions.

## 5 Concluding remarks

EOF has proven to be a practical fluid pumping method for transferring water and charged species through tiny slit channels or pores that our understanding of this unique phenomenon has been developing for more than two centuries. The key role in EOF plays by a charged layer of solution called the EDL that forms in the vicinity of a charged solid surface and consists of ordered water molecules and counter‐ions. In addition to the role of EDL, the intermediate domain has also demonstrated an interesting and unique impact on EOF. The relative size of the channel/porous media with respect to the thickness of EDL is also a critical parameter to determine EOF. This is the reason why the transport phenomena of water and ionic species at microscale and nanoscale are largely different. In this tutorial, we summarized the works that consider this effect for both straight channels and porous media.

The rising interest in employing EOF for emerging applications has encouraged scientists to conduct further theoretical and experimental studies. Among the various emerging research directions in this field, an interesting one involves multicomponent multiphase electrokinetic transport with an emphasis on transport in charged micro/nanochannel and porous media with broad applications in energy, biology, and environmental issues. Another interesting direction could be to study the impact of thermodynamical forces because of the concentration, viscosity, and temperature gradient on EOF through micro/nanochannels and porous media by taking into account the impact of these gradients on the local surface charge.

*This work is financially supported by the UTokyo‐Tsinghua Collaborative Research Fund, National Natural Science Foundation of China (No. 51766107), and the Tsinghua University Initiative Scientific Research Program*.

*The authors have declared no conflict of interest*.

## Data Availability

Data sharing is not applicable to this article as no new data were created or analyzed in this study.
